# Global, regional, and national burden of epilepsy, 1990–2016: a systematic analysis for the Global Burden of Disease Study 2016

**DOI:** 10.1016/S1474-4422(18)30454-X

**Published:** 2019-04

**Authors:** GBD 2016 Epilepsy Collaborators, Ettore Beghi, Giorgia Giussani, Foad Abd-Allah, Jemal Abdela, Ahmed Abdelalim, Haftom Niguse Abraha, Mina G. Adib, Sutapa Agrawal, Fares Alahdab, Ashish Awasthi, Yohanes Ayele, Miguel A Barboza, Abate Bekele Belachew, Belete Biadgo, Ali Bijani, Helen Bitew, Félix Carvalho, Yazan Chaiah, Ahmad Daryani, Huyen Phuc Do, Manisha Dubey, Aman Yesuf Yesuf Endries, Sharareh Eskandarieh, Andre Faro, Farshad Farzadfar, Seyed-Mohammad Fereshtehnejad, Eduarda Fernandes, Daniel Obadare Fijabi, Irina Filip, Florian Fischer, Abadi Kahsu Gebre, Afewerki Gebremeskel Tsadik, Teklu Gebrehiwo Gebremichael, Kebede Embaye Gezae, Maryam Ghasemi-Kasman, Kidu Gidey Weldegwergs, Meaza Girma Degefa, Elena V. Gnedovskaya, Tekleberhan B Hagos, Arvin Haj-Mirzaian, Arya Haj-Mirzaian, Hamid Yimam Hassen, Simon I Hay, Mihajlo Jakovljevic, Amir Kasaeian, Tesfaye Dessale Kassa, Yousef Saleh Khader, Ibrahim Khalil, Ejaz Ahmad Khan, Jagdish Khubchandani, Adnan Kisa, Kristopher J Krohn, Chanda Kulkarni, Yirga Legesse Nirayo, Mark T Mackay, Marek Majdan, Azeem Majeed, Treh Manhertz, Man Mohan Mehndiratta, Tesfa Mekonen, Hagazi Gebre Meles, Getnet Mengistu, Shafiu Mohammed, Mohsen Naghavi, Ali H Mokdad, Ghulam Mustafa, Seyed Sina Naghibi Irvani, Long Hoang Nguyen, Emma Nichols, Molly R Nixon, Felix Akpojene Ogbo, Andrew T Olagunju, Tinuke O Olagunju, Mayowa Ojo Owolabi, Michael R Phillips, Gabriel David Pinilla-Monsalve, Mostafa Qorbani, Amir Radfar, Anwar Rafay, Vafa Rahimi-Movaghar, Nickolas Reinig, Perminder S Sachdev, Hosein Safari, Saeed Safari, Saeid Safiri, Mohammad Ali Sahraian, Abdallah M. Samy, Shahabeddin Sarvi, Monika Sawhney, Masood A Shaikh, Mehdi Sharif, Gagandeep Singh, Mari Smith, Cassandra E I Szoeke, Rafael Tabarés-Seisdedos, Mohamad-Hani Temsah, Omar Temsah, Miguel Tortajada-Girbés, Bach Xuan Tran, Amanuel Amanuel Tesfay Tsegay, Irfan Ullah, Narayanaswamy Venketasubramanian, Ronny Westerman, Andrea Sylvia Winkler, Ebrahim M Yimer, Naohiro Yonemoto, Valery L. Feigin, Theo Vos, Christopher J L Murray

## Abstract

**Background:**

Seizures and their consequences contribute to the burden of epilepsy because they can cause health loss (premature mortality and residual disability). Data on the burden of epilepsy are needed for health-care planning and resource allocation. The aim of this study was to quantify health loss due to epilepsy by age, sex, year, and location using data from the Global Burden of Diseases, Injuries, and Risk Factors Study.

**Methods:**

We assessed the burden of epilepsy in 195 countries and territories from 1990 to 2016. Burden was measured as deaths, prevalence, and disability-adjusted life-years (DALYs; a summary measure of health loss defined by the sum of years of life lost [YLLs] for premature mortality and years lived with disability), by age, sex, year, location, and Socio-demographic Index (SDI; a compound measure of income per capita, education, and fertility). Vital registrations and verbal autopsies provided information about deaths, and data on the prevalence and severity of epilepsy largely came from population representative surveys. All estimates were calculated with 95% uncertainty intervals (UIs).

**Findings:**

In 2016, there were 45·9 million (95% UI 39·9–54·6) patients with all-active epilepsy (both idiopathic and secondary epilepsy globally; age-standardised prevalence 621·5 per 100 000 population; 540·1–737·0). Of these patients, 24·0 million (20·4–27·7) had active idiopathic epilepsy (prevalence 326·7 per 100 000 population; 278·4–378·1). Prevalence of active epilepsy increased with age, with peaks at 5–9 years (374·8 [280·1–490·0]) and at older than 80 years of age (545·1 [444·2–652·0]). Age-standardised prevalence of active idiopathic epilepsy was 329·3 per 100 000 population (280·3–381·2) in men and 318·9 per 100 000 population (271·1–369·4) in women, and was similar among SDI quintiles. Global age-standardised mortality rates of idiopathic epilepsy were 1·74 per 100 000 population (1·64–1·87; 1·40 per 100 000 population [1·23–1·54] for women and 2·09 per 100 000 population [1·96–2·25] for men). Age-standardised DALYs were 182·6 per 100 000 population (149·0–223·5; 163·6 per 100 000 population [130·6–204·3] for women and 201·2 per 100 000 population [166·9–241·4] for men). The higher DALY rates in men were due to higher YLL rates compared with women. Between 1990 and 2016, there was a non-significant 6·0% (−4·0 to 16·7) change in the age-standardised prevalence of idiopathic epilepsy, but a significant decrease in age-standardised mortality rates (24·5% [10·8 to 31·8]) and age-standardised DALY rates (19·4% [9·0 to 27·6]). A third of the difference in age-standardised DALY rates between low and high SDI quintile countries was due to the greater severity of epilepsy in low-income settings, and two-thirds were due to a higher YLL rate in low SDI countries.

**Interpretation:**

Despite the decrease in the disease burden from 1990 to 2016, epilepsy is still an important cause of disability and mortality. Standardised collection of data on epilepsy in population representative surveys will strengthen the estimates, particularly in countries for which we currently have no or sparse data and if additional data is collected on severity, causes, and treatment. Sizeable gains in reducing the burden of epilepsy might be expected from improved access to existing treatments in low-income countries and from the development of new effective drugs worldwide.

**Funding:**

Bill & Melinda Gates Foundation.

## Introduction

Epilepsy is a chronic disease of the CNS that affects individuals of all ages and has a worldwide distribution.^1^ The cardinal manifestations of epilepsy are epileptic seizures: that is, recurrent paroxysmal events characterised by stereotyped behavioural alterations reflecting the neural mechanisms involved in the epileptic process.^2^ Several diseases and injuries are implicated in the origin of epileptic seizures, with variable distribution in the world.^1^ Although many underlying disease mechanisms can lead to epilepsy, the cause of the disease is still unknown in about 50% of global cases.^1^ The diagnosis of epileptic seizures and epilepsy and ascertainment of the cause are difficult tasks, especially in low-income countries where socioeconomic and cultural constraints are obstacles to the recognition and acceptance of the disease.^3^ These limitations, along with the differing distribution of some environmental risk factors, are possible explanations for the heterogeneous frequency, course, and consequences of the disease in the world.^4^ Causes of secondary epilepsy include, among others, stroke, neurodegenerative disorders, infectious and inflammatory disorders, brain tumours, traumatic brain injuries, and congenital anomalies. However, these conditions were not considered risks in the Global Burden of Diseases, Injuries, and Risk Factors Study (GBD), but rather quantified as sequelae, or consequences, of the underlying causes of secondary epilepsy.

**Research in context**

**Evidence before this study**

We searched PubMed, without language restrictions, using the terms (((((epilepsy AND epidemiology) AND (“2011/01/01”[PDat] : “2015/12/31”[PDat]))) AND ((epilepsy AND epidemiology)))) to identify articles published between Jan 1, 2011, and Oct 17, 2016. The 2015 estimates from the Global Burden of Diseases, Injuries, and Risk Factors Study (GBD) suggested that epilepsy contributes to 0·5% of disability-adjusted life-years (DALYs) due to all diseases and injuries and 5·0% of DALYs attributable to neurological disorders. However, these aggregated data do not explain in detail the burden due to epilepsy by age, sex, location, and socioeconomic status. For GBD 2016, we estimated global, regional, and country-specific prevalence, and years lived with disability for active epilepsy from 1990 to 2016. 317 studies on the prevalence of epilepsy, 81 studies on incidence, and 23 studies on mortality were selected on the basis of the quality of evidence. Additional studies on the severity of the disease were selected for the calculation of disability weights (appendix).

**Added value of this study**

This systematic analysis for GBD 2016 is specifically aimed at informing epilepsy researchers and clinicians who might not have seen the general publications on this global public health resource. We present results on the burden of active idiopathic epilepsy (ie, epilepsy of genetic or unknown origin), exploring variation by age, sex, location, and year, as well as the association between epilepsy burden and development status of a country, as measured by the Socio-demographic Index (SDI), a compound measure of income per capita, education, and fertility. About the same number of people globally have idiopathic and secondary epilepsy. There is little variation by SDI in prevalence of idiopathic epilepsy, with rates in the five SDI quintiles of countries indistinguishable from the global age-standardised rate of 326·7 per 100 000 population (278·4–378·1) in 2016. Two-thirds of the gap in burden from idiopathic epilepsy between SDI quintiles is due to longer survival in people with epilepsy, and another third is from lesser severity of disease in high SDI quintile countries.

**Implications of all the available evidence**

From 1990 to 2016, significant changes in the burden of idiopathic epilepsy have been observed. These changes resulted from reduction in the case fatality rate and severity of disease rather than a change in prevalence. The low mortality rate in high SDI countries suggests that further gains can still be made in low and middle SDI countries because deaths from idiopathic epilepsy are largely avoidable with adequate management of the disease. Similarly, improved access to treatment can reduce burden by shifting people with epilepsy out of the state of recurrent seizures. The causes of secondary epilepsy are more amenable to prevention; although treatments can lead to the same successful control of seizures, they have less successful treatment overall because they do not address the often-severe comorbid disabilities from motor or intellectual impairments. In future GBDs, it would be advisable to explicitly aggregate all of the causes of secondary epilepsy that are currently estimated as sequelae (consequences) of underlying diseases and often in combined sequelae with motor, cognitive, or sensory impairments.

The recurrence of seizures and their physical and psychological consequences make epilepsy a burdensome neurological disorder. However, medical treatment of epilepsy with first-line antiepileptic drugs can render up to 70% of patients seizure free when adequately treated.^5^

According to a 2006 WHO report,^5^ 50 million people had epilepsy. The proportion of all disability-adjusted life-years (DALYs) attributed to epilepsy was 0·5%, but these findings are no longer comparable to current GBD estimates because of major differences in assumptions underpinning the DALY implemented after the WHO report. Comparative findings from different countries are scarce, and meta-analyses of prevalence or incidence studies^6^, ^7^, ^8^ do not take time of study into account, do not use predictive covariates, and are unable to predict estimates by country.

The GBD collaboration provides a systematic, comparable method of quantifying health loss in detail by disease, age, sex, year, location, and sociodemographic status. In a recent GBD report^9^ on the burden of neurological diseases, idiopathic epilepsy (ie, epilepsy of genetic origin or without a definite structural, metabolic, infective, or immune cause) accounted for 5·0% of total neurological DALYs and 1·3% of all deaths. Globally, idiopathic epilepsy ranked fifth among neurological disorders after stroke, migraine, dementia, and meningitis. Idiopathic epilepsy ranked second among neurological disorders in southern sub-Saharan Africa. When comparing the trends from 1990 to 2015, there was, however, a significant decrease in death and DALY rates attributable to idiopathic epilepsy, whereas the age-standardised prevalence rate remained stable globally. A more detailed assessment of DALYs in each country and over time was thus needed to verify if and to what extent the overall trends can be confirmed at the country level and, where available, at the regional level to identify discrepancies and, consequently, areas of intervention. A detailed account of the GBD estimates for epilepsy (including age-specific, sex-specific, time-specific, and geographical trends, and the sociodemographic context) was needed to make the information more accessible to researchers, clinicians, and planners of neurological services. We aimed to quantify health loss due to epilepsy by age, sex, year, and location using data from the GBD collaboration in 195 countries and territories from 1990 to 2016.

## Methods

### Overview

Details of the general GBD methodology are reported in the appendix, including the guiding principles to assess health loss, the selection and assessment of the quality of the data sources, the input data and modelling strategies to assess epilepsy mortality and impairment, and a list of key articles used for reference.

### Mortality

To assess premature mortality, we adhered to the underlying cause of death recorded in vital registration systems, as assigned by a physician on a death certificate. We made extensive corrections to cause of death data by redistributing deaths that were assigned to unspecified or intermediary causes on the basis of the International Classification of Diseases (ICD). The ICD-9 code for epilepsy is 345 and the ICD-10 codes for epilepsy are G40 and G41. Mortality from epilepsy was modelled with the Cause of Death Ensemble model, a tool developed for GBD cause of death analysis. The Cause of Death Ensemble model has the ability, through out-of-sample predictive validity testing, to determine which combination of covariates produces estimates that best cover the input data. This approach is different from analyses done to prove causality between a covariate or risk and an outcome. The data inputs in the model included 16 533 site-years of vital registration and 1093 site-years of verbal autopsy data; a site-year is a unique combination of location and calendar year. The model also included predictive covariates on pigs per capita and pig meat consumption as a proxy for neurocysticercosis infection,^10^, ^11^ systolic blood pressure, cholesterol,^12^ a measure of health-care access and quality,^13^ and a summary exposure measure of alcohol consumption.^14^ Additional details on calculations can be found in the GBD 2016 risk factor overview paper^15^ and in the appendix.

### Non-fatal disease modelling

The reference definition for epilepsy was based on the International League Against Epilepsy (ILAE) Guidelines for Epidemiologic Studies on Epilepsy,^16^ which defined an epilepsy case as someone with an active, recurrent condition of epileptic seizures (two or more) unprovoked by an immediate cause and who has had at least one epileptic seizure in the past 5 years regardless of antiepileptic drug treatment.

Systematic review of the literature yielded 319 unique sources of data on prevalence covering 20 of the 21 world regions, 82 unique sources of data on incidence covering 15 of the 21 world regions, and three unique sources of data on remission covering three of the 21 world regions. We also added 3 years of medical claims data from the USA. These data were defined in ICD-9 terms. The other sources of prevalence and incidence data were surveys stating case definitions independent of ICD codes.

Oceania was the only region for which no data were available. All data sources reported on all epilepsy (idiopathic and secondary combined). Where datapoints spanned an age group of more than 20 years, we split these data points into 5-year age bands by applying the age pattern from the USA, for which we had the most detailed data by age.

We modelled overall epilepsy prevalence and incidence using DisMod-MR 2.1, the Bayesian meta-regression tool developed for GBD. Study covariates were included to adjust US claims data to the reference epidemiological definition and to adjust studies with data on lifetime history of epilepsy to our active epilepsy definition. Additionally, we used a summary exposure measure of alcohol consumption and pig meat consumption per capita as predictive covariates on prevalence, as well as lag-distributed income as a predictive covariate on the excess mortality rate, or the excess rate of dying in cases of epilepsy in comparison with the general population.

### Idiopathic and secondary epilepsy

The overall epilepsy prevalence derived from this model was split into idiopathic epilepsy (ie, epilepsy due to a genetic cause or when diagnostic assessment did not reveal a causative factor) and secondary epilepsy (ie, epilepsy due to structural, metabolic, infective, or immune cause).

The term idiopathic is in accordance with the 1985 ILAE proposal for classification of epilepsies and epileptic syndromes.^17^ Although this terminology has been questioned in the latest ILAE classification of the epilepsies,^18^ we retained the old term because most of the epidemiological studies used as data sources adopted the old classification.

From a systematic review, we identified 89 unique sources of data reporting on the proportion of epilepsy that is due to genetic or unknown causes, covering 18 of the 21 world regions. We found, however, that not all the sources identified used advanced diagnostic methods (CT or MRI scans in addition to electroencephalograms) to diagnose secondary epilepsy, and that sources that did not use advanced diagnostic methods reported systematically lower proportions for secondary epilepsy. Therefore, we added a covariate to adjust the studies with less comprehensive diagnostic procedures to those that used all available methods to diagnose secondary epilepsy. We used these data in a linear mixed-effects model, with fixed effects on under-5 mortality rate, log-transformed pig meat consumption, access to sanitation, and the study quality covariate, as well as random effects for super-regions (ie, seven aggregates of 21 world regions defined in GBD). We obtained predictions for the proportion of idiopathic epilepsy from this model for every location and year, and applied them to the prevalence and incidence results of the DisMod-MR 2.1 model to calculate the prevalence and incidence of idiopathic epilepsy.

Secondary epilepsy was quantified as long-term consequences of meningitis, tetanus, malaria, cysticercosis, cystic echinococcosis, preterm birth complications, neonatal encephalopathy, neonatal sepsis, and neonatal haemolytic disease. Secondary epilepsy from other causes, such as brain cancer, traumatic brain injury, congenital anomalies, or stroke, was not quantified explicitly but assumed to be subsumed in the severity distributions and corresponding disability weights for those conditions.^19^

### Severity distributions and years lived with disability

Three health states were defined as sequelae of idiopathic epilepsy: severe epilepsy, defined as an average seizure frequency of more than or equal to once per month; less severe epilepsy with a seizure frequency of less than once a month, or no seizures in the past year while untreated but still fulfilling the criteria of active epilepsy; and seizure-free, treated epilepsy, defined as not having seizures in the past year while on treatment. All the data informing these splits were identified through systematic review. The data included 29 unique sources on the proportion of epilepsy that is severe, covering 12 of the 21 world regions; 68 unique sources on the proportion of epilepsy that is treated, covering 16 of the 21 world regions; and ten unique sources on the proportion of treated patients with epilepsy who do not have seizures, covering six of the 21 world regions. The distributions of cases across these three health states were quantified in three additional linear models. The first two splits, to derive the proportion of less severe epilepsy and then to calculate the proportion of less severe epilepsy that is treated, used linear models with a fixed effect on the index of health-care access and quality and random effects on super-region. To determine the proportion of treated epilepsy where patients had not reported seizures in the previous year, we ran a linear regression with a fixed effect on the index of health-care access and quality. We split out the prevalence and incidence for these categories by sequentially applying the proportions to the prevalence and incidence of idiopathic epilepsy. The final category of less severe epilepsy was calculated as the overall less severe epilepsy category, excluding treated epilepsy without seizures. Each one of the three severity categories has a specific disability weight, and years lived with disability (YLDs) were calculated as prevalence multiplied by the category-specific disability weight. Further details on the methodology are provided in the appendix.

### Socio-demographic Index

The frequency and severity of epilepsy were also assessed with reference to the Socio-demographic Index (SDI), a composite measure developed to provide a comparable metric of overall socioeconomic development in each country and represented by the gross domestic product per capita, the average years of education in the population older than 15 years of age, and the total fertility rate.^20^

### Risk estimation

Alcohol use was the only risk of the 84 risks included in GBD 2016 for which there was deemed to be sufficient evidence for a causal relationship with idiopathic epilepsy as an outcome. Population-attributable fractions were estimated using data for exposure, relative risk, and a theoretical-minimum exposure level. Additional details on calculations can be found in the GBD 2016 risk factor overview paper.^15^

### Compilation of results

Years of life lost (YLLs) were calculated by multiplying the number of deaths at each age group by the remaining life expectancy at that age, which was derived from the GBD standard life table.^21^ DALYs were then calculated by summing YLLs and YLDs. We propagated uncertainty at each step of the analytical process by sampling 1000 draws at each computational step. Uncertainty intervals (UIs) were defined as the 25th and 975th values of the ordered draws. The term rate was used to indicate the number of cases per 100 000 population, in keeping with the other GBD reports. Differences in rates and counts between 1990 and 2016 are presented as significant if more than 950 of 1000 draws were all negative, or all positive. The study is compliant with the Guidelines for Accurate and Transparent Health Estimates Reporting (appendix).

### Role of the funding source

The funder of the study had no role in study design, data collection, data analysis, data interpretation, or the writing of the report. All authors had full access to the data in the study and had final responsibility for the decision to submit for publication.

## Results

In 2016, there were 45·9 million (95% UI 39·9–54·6) individuals with active epilepsy of idiopathic or secondary nature globally. Of these individuals, 24·0 million (20·4–27·7) had active idiopathic epilepsy. There were 126 055 epilepsy-related deaths (118 632–135 517) and 13·5 million DALYs (11·0–16·5; table 1), and there were 5·9 million (5·6–6·4) YLLs and 7·5 million (5·1–10·5) YLDs. Idiopathic epilepsy accounted for 0·23% (0·22–0·25) of deaths and 0·56% (0·48–0·66) of DALYs from all causes. Global age-standardised mortality rates of idiopathic epilepsy were 1·74 per 100 000 population (1·64–1·87; 1·40 per 100 000 population [1·23–1·54] for women and 2·09 per 100 000 population [1·96–2·25] for men).

**Table 1:** Deaths, prevalence, and DALYs for idiopathic epilepsy in 2016, and percentage change in age-standardised rates by location

	**Deaths**		**Prevalence**		**DALYs**	
	2016 counts	Percentage change in age-standardised rates, 1990–2016	2016 counts	Percentage change in age-standardised rates 1990–2016	2016 counts	Percentage change in age-standardised rates, 1990–2016
**Global**	**126 055 (118 632 to 135 517)**	**−24·5% (−31·8 to −10·8)**	**23 962 448 (20 401 828 to 27 737 043)**	**6·0% (−4·0 to 16·7)**	**13 492 251 (11 014 685 to 16 503 078)**	**−19·4% (−27·6 to −9·0)**
High SDI	12 744 (12 203 to 13 558)	−2·7% (−7·2 to 4·8)	3 357 612 (2 678 423 to 4 025 445)	10·6% (−11·6 to 39·1)	1 187 528 (908 278 to 1 533 569)	−7·6% (−23·7 to 11·3)
High-middle SDI	10 938 (10 194 to 12 003)	−39·2% (−45·8 to −27·6)	3 374 755 (2 637 686 to 4 109 936)	3·0% (−19·6 to 34·0)	1 473 794 (1 129 349 to 1 911 994)	−27·1% (−41·2 to −10·6)
Middle SDI	36 153 (34 422 to 38 680)	−33·7% (−38·8 to −22·5)	7 864 730 (6 633 313 to 9 248 943)	8·9% (−5·4 to 26·5)	4 145 107 (3 342 749 to 5 089 933)	−23·0% (−31·3 to −13·6)
Low-middle SDI	48 802 (43 507 to 55 892)	−30·6% (−40·3 to −13·5)	6 832 353 (5 380 975 to 8 350 595)	1·6% (−19·3 to 26·1)	4 753 027 (3 884 858 to 5 876 862)	−26·3% (−38·1 to −10·0)
Low SDI	17 360 (15 695 to 19 187)	−12·1% (−23·3 to 10·3)	2 479 921 (1 819 635 to 3 214 747)	4·5% (−24·9 to 45·4)	1 914 283 (1 543 598 to 2 399 375)	−12·7% (−28·1 to 10·0)
**High-income North America**	**2391 (2309 to 2475)**	**−12·3% (−15·7 to −8·9)**	**1 266 669 (990 026 to 1 532 133)**	**10·7% (−13·5 to 44·8)**	**403 536 (290 787 to 546 430)**	**−3·0% (−23·2 to 23·5)**
Canada	314 (288 to 345)	−28·9% (−35·2 to −21·7)	84 838 (21 473 to 147 748)	2·0% (−78·3 to 361·2)	30 135 (14 286 to 58 457)	−17·0% (−67·4 to 105·8)
Greenland	2 (1 to 2)	−29·9% (−46·7 to −6·0)	191 (46 to 352)	−11·5% (−81·3 to 332·5)	110 (64 to 181)	−29·1% (−65·5 to 44·6)
USA	2076 (2002 to 2150)	−9·7% (−13·2 to −6·0)	1 181 207 (927 479 to 1 429 263)	11·4% (−14·3 to 47·4)	373 183 (265 183 to 509 146)	−1·7% (−23·1 to 26·6)
**Australasia**	**346 (319 to 375)**	**−15·1% (−22·6 to −7·0)**	**65 797 (24 377 to 109 378)**	**−1·5% (−67·2 to 191·5)**	**27 725 (16 880 to 47 808)**	**−18·3% (−56·9 to 55·2)**
Australia	293 (268 to 320)	−11·8% (−20·6 to −2·3)	55 674 (14 413 to 97 939)	0·4% (−75·5 to 297·9)	23 141 (12 914 to 42 582)	−16·0% (−60·8 to 84·8)
New Zealand	53 (47 to 60)	−27·6% (−36·7 to −16·7)	10 123 (2476 to 17 892)	−10·2% (−81·3 to 252·2)	4584 (2628 to 8120)	−26·8% (−62·7 to 49·0)
**High-income Asia Pacific**	**1239 (1076 to 1427)**	**−18·6% (−30·8 to −3·0)**	**364 894 (264 943 to 478 045)**	**−0·4% (−30·7 to 48·0)**	**124 775 (90 045 to 171 858)**	**−21·7% (−43·9 to 10·4)**
Brunei	8 (6 to 10)	−13·7% (−35·5 to 17·4)	1234 (346 to 2090)	−15·7% (−79·5 to 258·1)	791 (473 to 1262)	−18·8% (−57·8 to 70·1)
Japan	681 (640 to 752)	−10·3% (−16·1 to −0·8)	247 249 (196 541 to 306 896)	4·3% (−16·3 to 30·9)	75 644 (54 202 to 102 261)	−11·4% (−29·4 to 9·5)
Singapore	16 (13 to 20)	−28·4% (−43·1 to −9·0)	8590 (2196 to 15 222)	1·9% (−73·7 to 272·6)	2666 (1016 to 5160)	−24·6% (−72·5 to 99·8)
South Korea	535 (389 to 705)	−30·0% (−49·6 to −5·1)	107 821 (26 860 to 188 521)	−10·0% (−79·0 to 299·2)	45 674 (23 369 to 79 779)	−35·8% (−71·4 to 47·8)
**Western Europe**	**7842 (7407 to 8435)**	**10·7% (4·4 to 20·0)**	**1 525 168 (1 026 982 to 2 010 466)**	**12·0% (−25·7 to 74·4)**	**553 078 (405 740 to 765 899)**	**−5·7% (−33·5 to 32·3)**
Andorra	1 (1 to 1)	−8·8% (−35·0 to 28·6)	237 (61 to 411)	−8·9% (−77·0 to 347·6)	85 (44 to 157)	−18·1% (−64·8 to 103·3)
Austria	88 (78 to 99)	−12·1% (−23·0 to 0·4)	23 424 (4812 to 42 084)	0·4% (−79·1 to 376·7)	8103 (3579 to 16 383)	−18·6% (−68·8 to 100·3)
Belgium	282 (248 to 321)	45·4% (27·5 to 66·5)	41 917 (10 360 to 74 029)	12·5% (−76·6 to 397·1)	16 007 (8515 to 28 340)	3·3% (−58·6 to 163·4)
Cyprus	8 (7 to 9)	−30·0% (−40·6 to −12·9)	2314 (513 to 4097)	−0·5% (−77·6 to 354·5)	811 (354 to 1580)	−21·5% (−69·8 to 105·5)
Denmark	88 (75 to 105)	38·6% (14·6 to 70·1)	14 926 (4016 to 26 083)	9·2% (−73·7 to 380·3)	6187 (3424 to 10 682)	4·1% (−54·7 to 144·9)
Finland	77 (68 to 90)	−13·1% (−25·8 to 5·7)	14 276 (3232 to 24 707)	2·4% (−79·8 to 416·5)	5809 (3032 to 10 562)	−17·1% (−63·8 to 87·3)
France	1703 (1542 to 1879)	4·9% (−5·4 to 16·7)	304 489 (76 321 to 531 242)	11·7% (−75·2 to 413·6)	108 670 (49 840 to 203 680)	−7·9% (−64·4 to 142·6)
Germany	2308 (2062 to 2583)	51·6% (34·5 to 71·9)	429 396 (109 560 to 761 662)	51·9% (−66·6 to 637·2)	153 712 (70 909 to 296 374)	24·0% (−52·6 to 235·5)
Greece	75 (68 to 84)	−9·7% (−20·0 to 2·0)	28 256 (6981 to 49 284)	−0·2% (−77·4 to 315·3)	8753 (3642 to 17 787)	−14·2% (−69·4 to 160·2)
Iceland	4 (4 to 5)	45·8% (29·5 to 64·0)	908 (250 to 1545)	3·7% (−74·0 to 297·5)	319 (166 to 608)	−0·2% (−59·9 to 165·4)
Ireland	60 (51 to 70)	−12·8% (−26·0 to 2·3)	13 153 (3455 to 23 515)	1·0% (−75·6 to 379·3)	5231 (2689 to 9628)	−14·4% (−63·9 to 107·9)
Israel	93 (77 to 111)	20·8% (−3·3 to 47·2)	20 176 (5762 to 33 993)	6·8% (−72·3 to 304·2)	8069 (4125 to 14 281)	−1·1% (−56·0 to 132·1)
Italy	730 (648 to 826)	11·4% (−1·2 to 28·2)	163 995 (39 382 to 287 959)	−14·1% (−81·3 to 300·9)	52 778 (23 068 to 104 089)	−23·8% (−75·4 to 131·9)
Luxembourg	11 (10 to 13)	−0·9% (−15·2 to 16·2)	1813 (463 to 3227)	−0·9% (−78·6 to 342·7)	719 (401 to 1285)	−18·3% (−65·3 to 96·4)
Malta	4 (3 to 5)	22·0% (−0·6 to 47·7)	1096 (277 to 1967)	7·4% (−75·2 to 368·2)	385 (172 to 751)	0·9% (−65·8 to 188·5)
Netherlands	268 (240 to 299)	2·0% (−10·4 to 15·2)	51 678 (12 813 to 90 495)	6·2% (−76·8 to 353·4)	18 951 (9236 to 34 557)	−8·1% (−61·8 to 126·9)
Norway	79 (70 to 90)	−17·3% (−28·7 to −3·8)	18 119 (4897 to 30 666)	4·8% (−74·1 to 339·6)	6549 (3270 to 12 227)	−16·6% (−65·3 to 100·0)
Portugal	169 (152 to 190)	−15·9% (−25·7 to −4·1)	24 437 (5590 to 43 209)	3·0% (−78·8 to 345·6)	10 049 (5144 to 17 829)	−27·0% (−66·9 to 64·3)
Spain	452 (405 to 515)	1·7% (−10·0 to 20·1)	105 520 (26 203 to 187 007)	8·2% (−76·7 to 357·3)	33 847 (15 438 to 67 771)	−14·5% (−70·6 to 131·2)
Sweden	111 (98 to 126)	−20·5% (−30·5 to −8·9)	22 010 (8669 to 35 691)	−8·7% (−67·2 to 164·7)	8370 (4774 to 14 168)	−22·9% (−59·0 to 44·7)
Switzerland	107 (84 to 134)	−40·3% (−53·9 to −22·9)	19 797 (4601 to 35 373)	−8·0% (−80·3 to 272·2)	6923 (3293 to 13 240)	−33·1% (−72·5 to 51·0)
UK	1123 (1087 to 1172)	−6·9% (−10·5 to −2·3)	221 727 (182 221 to 261 683)	−0·3% (−14·8 to 16·3)	92 400 (74 455 to 114 293)	−12·3% (−22·0 to −1·3)
**Southern Latin America**	**592 (522 to 670)**	**−19·7% (−29·4 to −8·2)**	**184 053 (84 034 to 283 046)**	**1·8% (−57·6 to 140·4)**	**78 384 (44 592 to 121 412)**	**−17·9% (−55·4 to 49·3)**
Argentina	284 (260 to 312)	−20·6% (−28·7 to −11·1)	101 909 (27 878 to 174 824)	4·2% (−73·2 to 370·6)	44 400 (19 443 to 83 007)	−13·2% (−64·2 to 116·1)
Chile	256 (198 to 327)	−25·5% (−43·0 to −3·2)	71 432 (16 341 to 124 915)	−2·6% (−81·3 to 264·9)	29 085 (12 901 to 52 219)	−25·9% (−72·5 to 73·8)
Uruguay	52 (47 to 56)	−3·1% (−14·0 to 8·1)	10 704 (2755 to 18 961)	3·7% (−75·8 to 336·2)	4897 (2428 to 8783)	−11·7% (−61·4 to 101·5)
**Eastern Europe**	**1869 (1512 to 2347)**	**−40·4% (−51·6 to −26·3)**	**431 632 (174 470 to 721 540)**	**−7·0% (−64·1 to 149·9)**	**204 537 (120 880 to 332 781)**	**−31·5% (−63·2 to 23·2)**
Belarus	141 (110 to 188)	−24·3% (−41·9 to 4·0)	21 987 (4977 to 41 306)	−0·6% (−79·8 to 419·2)	12 079 (6723 to 20 740)	−22·4% (−62·5 to 49·0)
Estonia	33 (28 to 41)	−1·1% (−19·8 to 23·9)	3813 (763 to 7187)	4·8% (−84·4 to 362·2)	2186 (1365 to 3559)	−12·9% (−55·8 to 68·8)
Latvia	40 (33 to 48)	21·0% (−1·0 to 48·1)	4820 (939 to 9715)	−1·0% (−80·7 to 404·5)	2814 (1745 to 4569)	−3·0% (−52·0 to 109·0)
Lithuania	63 (56 to 72)	−12·8% (−22·8 to 0·1)	7858 (1788 to 15 017)	0·2% (−79·9 to 371·3)	4587 (2847 to 7865)	−15·0% (−56·3 to 70·0)
Moldova	68 (59 to 79)	−37·9% (−47·5 to −25·6)	7935 (1988 to 15 121)	−12·3% (−80·5 to 427·0)	5216 (3364 to 8342)	−36·9% (−64·6 to 15·7)
Russia	882 (636 to 1209)	−54·9% (−67·9 to −37·7)	288 899 (57 036 to 544 297)	−8·7% (−83·1 to 404·8)	120 960 (51 274 to 236 133)	−38·4% (−76·1 to 50·2)
Ukraine	642 (464 to 886)	−10·9% (−36·6 to 25·5)	96 320 (23 225 to 180 766)	−3·6% (−81·4 to 425·8)	56 695 (31 720 to 96 555)	−14·6% (−58·1 to 83·5)
**Central Europe**	**1848 (1715 to 2013)**	**−14·6% (−21·3 to −4·5)**	**358 718 (226 469 to 506 369)**	**7·2% (−35·7 to 81·3)**	**159 221 (114 662 to 219 235)**	**−18·2% (−41·2 to 14·8)**
Albania	53 (45 to 64)	−11·3% (−25·4 to 8·7)	11 093 (2563 to 21 126)	1·2% (−78·7 to 409·2)	5572 (3040 to 9683)	−12·1% (−56·6 to 86·2)
Bosnia and Herzegovina	67 (56 to 80)	−39·8% (−50·9 to −26·5)	12 234 (2827 to 22 374)	−1·6% (−79·3 to 393·9)	5630 (3049 to 10 416)	−33·5% (−68·7 to 39·4)
Bulgaria	104 (87 to 124)	−12·2% (−28·3 to 4·9)	22 376 (4886 to 42 511)	6·3% (−76·1 to 457·1)	10 207 (5062 to 19 113)	−12·1% (−63·3 to 96·7)
Croatia	86 (74 to 100)	4·6% (−12·8 to 26·4)	12 506 (2506 to 24 565)	−4·3% (−81·1 to 389·9)	5826 (3282 to 10 581)	−16·0% (−62·5 to 88·9)
Czech Republic	136 (123 to 156)	−26·2% (−34·8 to −13·0)	35 197 (7181 to 67 984)	6·4% (−79·6 to 483·7)	12 989 (6034 to 25 006)	−23·1% (−70·2 to 94·3)
Hungary	107 (92 to 124)	−47·2% (−56·5 to −36·4)	30 177 (5789 to 60 440)	−4·4% (−81·6 to 382·7)	11 631 (5288 to 24 794)	−35·3% (−75·3 to 58·2)
Macedonia	25 (22 to 27)	−32·3% (−41·4 to −22·9)	6373 (1486 to 11 683)	7·2% (−77·8 to 429·8)	2649 (1253 to 4923)	−23·1% (−70·2 to 79·9)
Montenegro	5 (4 to 5)	−15·1% (−26·9 to −0·1)	1759 (374 to 3471)	6·3% (−80·7 to 475·9)	626 (252 to 1328)	−12·0% (−69·5 to 164·0)
Poland	690 (599 to 789)	22·5% (5·1 to 45·3)	107 139 (23 217 to 204 047)	19·6% (−79·0 to 515·2)	50 967 (28 183 to 91 348)	−1·6% (−58·5 to 138·3)
Romania	266 (235 to 304)	−33·6% (−42·2 to −24·0)	58 789 (12 162 to 113 336)	0·5% (−78·7 to 569·3)	26 202 (12 902 to 49 204)	−28·9% (−70·4 to 62·6)
Serbia	160 (146 to 177)	−14·3% (−25·2 to −1·6)	34 026 (8199 to 64 275)	16·9% (−76·3 to 496·4)	14 739 (7484 to 28 301)	−10·2% (−62·6 to 111·3)
Slovakia	126 (107 to 150)	−0·9% (−20·9 to 28·9)	20 226 (4646 to 38 630)	10·2% (−77·4 to 484·7)	9933 (5543 to 17 477)	−6·9% (−58·0 to 110·3)
Slovenia	23 (20 to 27)	−53·1% (−61·4 to −42·4)	6822 (1470 to 12 806)	−2·6% (−79·6 to 435·3)	2248 (947 to 4645)	−36·0% (−76·8 to 79·3)
**Central Asia**	**3225 (2885 to 3659)**	**46·7% (31·6 to 66·6)**	**350 725 (208 401 to 511 731)**	**18·0% (−33·0 to 107·2)**	**287 949 (226 678 to 372 275)**	**23·4% (−6·4 to 68·1)**
Armenia	17 (15 to 20)	−63·6% (−68·6 to −57·5)	9642 (2147 to 18 659)	7·4% (−80·0 to 426·4)	3662 (1328 to 7284)	−33·7% (−78·5 to 69·4)
Azerbaijan	260 (199 to 339)	11·3% (−14·8 to 46·7)	38 421 (9 489 to 70 541)	25·5% (−72·4 to 632·8)	26 774 (16 136 to 43 259)	6·8% (−45·2 to 110·0)
Georgia	40 (33 to 48)	−52·6% (−61·5 to −42·8)	13 424 (2705 to 25 872)	4·5% (−79·6 to 516·4)	6115 (2517 to 11 647)	−28·7% (−72·9 to 60·9)
Kazakhstan	316 (239 to 452)	−2·9% (−26·8 to 38·7)	61 571 (13 281 to 121 213)	6·7% (−77·3 to 436·4)	36 654 (18 495 to 65 154)	−3·3% (−57·4 to 111·4)
Kyrgyzstan	195 (170 to 228)	3·6% (−10·1 to 21·7)	19 025 (4001 to 38 756)	−10·6% (−82·7 to 339·0)	16 634 (11 127 to 25 347)	−10·5% (−50·5 to 61·6)
Mongolia	83 (64 to 100)	30·9% (−4·9 to 64·4)	11 107 (2543 to 20 819)	43·6% (−69·2 to 612·1)	8087 (5014 to 12 661)	22·8% (−37·7 to 151·2)
Tajikistan	398 (318 to 514)	18·0% (−10·9 to 53·9)	32 028 (6802 to 63 049)	−12·3% (−83·8 to 301·7)	34 022 (23 148 to 48 864)	−1·3% (−43·4 to 69·4)
Turkmenistan	128 (103 to 156)	2·5% (−17·3 to 26·4)	19 295 (4266 to 38 101)	18·0% (−79·9 to 609·3)	14 218 (8351 to 23 453)	1·6% (−51·6 to 111·9)
Uzbekistan	1789 (1549 to 2031)	95·6% (69·9 to 125·7)	146 213 (37 541 to 274 793)	34·2% (−69·2 to 435·2)	141 783 (99 492 to 208 946)	54·4% (−9·3 to 162·2)
**Central Latin America**	**3858 (3612 to 4152)**	**−28·1% (−32·5 to −23·6)**	**1 361 130 (1 015 253 to 1 743 102)**	**−6·2% (−31·2 to 27·6)**	**578 968 (422 303 to 780 317)**	**−25·4% (−40·2 to −6·6)**
Colombia	546 (475 to 634)	−30·5% (−40·3 to −16·5)	259 714 (59 331 to 473 541)	−3·8% (−79·7 to 313·9)	97 000 (38 597 to 187 054)	−27·3% (−73·0 to 84·6)
Costa Rica	54 (48 to 60)	−34·6% (−41·9 to −26·6)	24 952 (7024 to 42 217)	5·3% (−75·9 to 366·3)	8491 (3561 to 16 526)	−18·0% (−70·5 to 121·8)
El Salvador	64 (55 to 75)	−35·8% (−46·5 to −17·9)	30 514 (7543 to 51 484)	10·4% (−76·0 to 368·3)	11 761 (4874 to 22 281)	−23·0% (−71·3 to 83·9)
Guatemala	360 (280 to 449)	−16·0% (−36·6 to 7·3)	66 241 (17 849 to 121 445)	4·6% (−74·5 to 403·8)	41 017 (23 936 to 68 350)	−16·2% (−57·4 to 65·5)
Honduras	202 (144 to 284)	−22·9% (−44·2 to 5·9)	42 165 (10 887 to 75 230)	−2·4% (−78·7 to 330·6)	23 578 (12 269 to 39 543)	−20·8% (−65·5 to 70·6)
Mexico	1989 (1903 to 2088)	−31·8% (−34·7 to −28·3)	727 028 (558 990 to 909 062)	−8·7% (−31·6 to 23·5)	306 444 (223 558 to 406 513)	−27·9% (−42·5 to −10·3)
Nicaragua	77 (64 to 95)	−35·9% (−47·1 to −21·3)	29 296 (8468 to 49 040)	−2·8% (−76·5 to 303·2)	12 233 (5840 to 21 938)	−30·5% (−70·0 to 56·1)
Panama	54 (46 to 64)	−10·3% (−25·6 to 8·8)	16 256 (3819 to 29 184)	4·7% (−75·8 to 367·2)	6927 (3242 to 12 790)	−9·7% (−63·2 to 116·6)
Venezuela	513 (411 to 674)	−19·8% (−37·1 to 3·3)	164 964 (44 841 to 291 195)	−6·0% (−77·9 to 313·9)	71 516 (32 568 to 130 833)	−19·1% (−66·7 to 106·4)
**Andean Latin America**	**605 (541 to 699)**	**−57·3% (−63·9 to −47·3)**	**261 097 (115 128 to 404 582)**	**−5·3% (−58·0 to 110·2)**	**104 271 (60 985 to 172 104)**	**−40·6% (−66·1 to 4·3)**
Bolivia	172 (138 to 212)	−51·3% (−63·8 to −31·8)	32 455 (6889 to 64 243)	−25·0% (−84·6 to 268·8)	18 739 (10 233 to 32 312)	−47·9% (−73·9 to 4·0)
Ecuador	248 (222 to 277)	−54·6% (−59·9 to −49·0)	88 466 (20 015 to 156 608)	−8·4% (−80·5 to 319·4)	38 919 (17 712 to 70 540)	−38·8% (−74·8 to 34·2)
Peru	185 (144 to 265)	−64·8% (−73·8 to −44·5)	140 175 (32 569 to 254 196)	2·6% (−79·1 to 355·7)	46 613 (16 342 to 97 928)	−39·8% (−80·6 to 58·6)
**Caribbean**	**770 (664 to 898)**	**−30·5% (−39·7 to −18·8)**	**136 726 (82 218 to 192 747)**	**−7·5% (−48·4 to 59·4)**	**75 882 (54 358 to 102 602)**	**−26·2% (−46·9 to 1·9)**
Antigua and Barbuda	3 (3 to 3)	−25·3% (−36·5 to −11·4)	428 (116 to 720)	−5·6% (−77·8 to 327·8)	242 (144 to 383)	−19·8% (−60·1 to 52·6)
The Bahamas	6 (5 to 7)	−30·2% (−40·5 to −17·8)	1375 (308 to 2506)	−8·1% (−80·6 to 282·8)	663 (338 to 1162)	−26·2% (−69·2 to 60·6)
Barbados	6 (6 to 7)	−23·3% (−34·2 to −9·7)	1075 (284 to 1863)	0·1% (−75·6 to 340·1)	511 (279 to 876)	−17·5% (−60·9 to 77·6)
Belize	7 (6 to 8)	0·6% (−16·1 to 21·1)	1228 (276 to 2240)	6·1% (−78·0 to 384·2)	724 (410 to 1158)	−8·4% (−54·3 to 80·1)
Bermuda	1 (1 to 1)	−57·6% (−64·9 to −48·6)	270 (71 to 479)	−12·6% (−80·4 to 304·4)	86 (35 to 168)	−44·2% (−79·4 to 42·1)
Cuba	95 (85 to 106)	−34·1% (−41·7 to −24·0)	33 247 (8371 to 57 987)	−0·8% (−76·3 to 312·7)	12 063 (5352 to 22 358)	−23·4% (−70·1 to 78·6)
Dominica	3 (3 to 4)	−12·4% (−24·8 to 3·3)	302 (74 to 560)	−2·2% (−77·6 to 340·5)	217 (137 to 331)	−9·3% (−48·3 to 63·3)
Dominican Republic	101 (84 to 123)	−41·9% (−52·7 to −27·3)	31 056 (7542 to 58 227)	4·9% (−78·7 to 450·1)	13 842 (6180 to 25 438)	−27·6% (−71·2 to 71·8)
Grenada	3 (2 to 4)	−3·1% (−22·7 to 19·1)	416 (102 to 754)	3·0% (−76·8 to 352·5)	261 (157 to 425)	−6·6% (−52·6 to 80·4)
Guyana	23 (19 to 26)	−24·7% (−36·9 to −11·0)	2827 (693 to 5043)	−1·1% (−76·9 to 336·1)	2013 (1276 to 3014)	−20·5% (−55·1 to 40·1)
Haiti	345 (235 to 479)	−29·6% (−47·7 to 4·1)	24 380 (3980 to 56 714)	−15·2% (−88·4 to 494·0)	27 515 (16 250 to 44 192)	−32·9% (−61·9 to 30·9)
Jamaica	52 (42 to 66)	−28·0% (−43·6 to −5·5)	11 018 (2808 to 19 011)	−3·6% (−75·9 to 283·8)	5336 (2746 to 9348)	−22·9% (−63·2 to 62·5)
Puerto Rico	59 (53 to 67)	−48·4% (−56·0 to −39·9)	14 581 (3851 to 25 955)	−15·3% (−77·7 to 243·8)	5371 (2676 to 9620)	−39·7% (−73·8 to 34·9)
Saint Lucia	5 (5 to 6)	−35·0% (−42·7 to −27·0)	705 (166 to 1278)	−7·1% (−80·1 to 405·7)	423 (256 to 683)	−28·6% (−62·3 to 37·6)
Saint Vincent and the Grenadines	4 (3 to 4)	−0·2% (−14·9 to 15·7)	428 (99 to 764)	5·0% (−76·2 to 336·0)	291 (181 to 457)	−4·4% (−47·1 to 77·4)
Suriname	15 (14 to 18)	−13·8% (−25·7 to 0·6)	1920 (403 to 3682)	−10·0% (−83·5 to 280·3)	1275 (777 to 2038)	−21·0% (−57·9 to 50·0)
Trinidad and Tobago	39 (34 to 45)	−23·2% (−34·6 to −10·7)	5970 (1546 to 10 731)	−5·3% (−77·4 to 365·2)	3389 (2012 to 5509)	−19·1% (−57·9 to 57·5)
Virgin Islands	2 (2 to 2)	−25·0% (−43·3 to −2·9)	440 (98 to 766)	−3·5% (−80·4 to 326·5)	176 (81 to 322)	−20·8% (−66·7 to 83·7)
**Tropical Latin America**	**2583 (2436 to 2770)**	**−9·1% (−16·3 to −2·8)**	**848 153 (609 618 to 1 089 267)**	**−4·9% (−35·4 to 38·3)**	**348 742 (252 247 to 458 694)**	**−19·6% (−39·2 to 5·5)**
Brazil	2495 (2346 to 2680)	−9·3% (−16·6 to −3·0)	825 349 (581 035 to 1 060 081)	−5·2% (−35·4 to 40·3)	337 631 (242 624 to 446 686)	−19·9% (−40·1 to 6·4)
Paraguay	88 (76 to 101)	−4·7% (−21·1 to 16·1)	22 804 (5419 to 42 059)	7·8% (−78·1 to 442·9)	11 111 (5491 to 20 340)	−5·7% (−61·4 to 128·1)
**East Asia**	**13 613 (12 795 to 15 210)**	**−55·8% (−59·9 to −47·8)**	**3 393 239 (2 737 873 to 4 036 642)**	**5·8% (−15·8 to 33·3)**	**1 659 992 (1 289 718 to 2 078 067)**	**−39·5% (−49·4 to −27·8)**
China	12 779 (11 966 to 14 361)	−57·3% (−61·5 to −49·3)	3 261 138 (2 613 977 to 3 892 677)	5·6% (−16·7 to 34·4)	1 582 425 (1 230 751 to 1 994 093)	−40·7% (−50·7 to −28·7)
North Korea	453 (371 to 555)	13·7% (−14·3 to 43·8)	58 159 (17 262 to 88 927)	3·2% (−71·2 to 297·4)	43 716 (28 086 to 63 371)	4·6% (−39·9 to 94·0)
Taiwan (province of China)	381 (328 to 440)	−32·5% (−45·5 to −10·8)	73 942 (24 580 to 112 971)	17·5% (−65·4 to 281·0)	33 851 (17 042 to 54 509)	−14·5% (−59·5 to 70·9)
**Southeast Asia**	**9274 (8359 to 9975)**	**−17·1% (−27·3 to −1·0)**	**2 476 546 (1 936 233 to 3 038 069)**	**15·1% (−11·3 to 46·8)**	**1 339 006 (1 008 109 to 1 733 718)**	**−6·1% (−23·3 to 15·3)**
Cambodia	260 (212 to 314)	−18·4% (−33·6 to 11·5)	58 844 (17 847 to 97 084)	21·8% (−65·4 to 455·8)	36 251 (19 511 to 58 011)	−7·7% (−54·5 to 91·0)
Indonesia	3260 (2880 to 3616)	−11·4% (−24·8 to 4·8)	961 337 (745 715 to 1 181 120)	20·5% (−8·3 to 62·4)	531 484 (391 440 to 694 387)	−1·7% (−22·1 to 24·5)
Laos	134 (96 to 178)	−19·0% (−37·8 to 16·6)	19 476 (4652 to 34 802)	23·2% (−76·4 to 558·9)	16 126 (8338 to 24 624)	−8·7% (−54·2 to 84·0)
Malaysia	253 (228 to 284)	−22·3% (−34·1 to −4·9)	127 814 (50 455 to 188 070)	13·5% (−58·2 to 230·4)	54 867 (26 378 to 94 205)	−6·1% (−57·7 to 101·9)
Maldives	4 (4 to 6)	−60·9% (−75·7 to −23·0)	1607 (600 to 2315)	−11·5% (−67·5 to 139·3)	693 (339 to 1158)	−50·0% (−76·3 to 9·3)
Mauritius	28 (24 to 33)	−9·6% (−23·2 to 5·8)	6675 (1953 to 9616)	11·7% (−66·7 to 216·8)	3376 (1726 to 5297)	−3·7% (−52·8 to 77·9)
Myanmar	955 (813 to 1175)	−21·7% (−37·2 to 3·4)	196 070 (60 852 to 313 438)	13·2% (−67·1 to 309·7)	123 865 (66 239 to 193 398)	−9·3% (−54·6 to 72·9)
Philippines	819 (701 to 948)	−14·7% (−27·2 to −1·1)	345 441 (95 379 to 570 826)	8·0% (−71·2 to 270·3)	176 577 (75 952 to 305 773)	−3·7% (−59·3 to 113·4)
Sri Lanka	318 (254 to 401)	−18·0% (−35·8 to 5·0)	124 196 (54 642 to 164 196)	15·5% (−48·0 to 155·5)	51 538 (27 088 to 81 058)	−6·5% (−52·0 to 76·9)
Seychelles	1 (1 to 1)	−31·9% (−46·6 to −10·1)	444 (156 to 649)	4·4% (−65·4 to 247·2)	199 (93 to 333)	−16·9% (−64·1 to 77·2)
Thailand	693 (598 to 792)	70·4% (25·2 to 110·8)	279 933 (95 172 to 405 229)	22·5% (−59·5 to 223·8)	117 533 (52 264 to 199 495)	12·8% (−50·7 to 160·4)
Timor-Leste	13 (9 to 18)	−30·1% (−58·4 to 18·6)	3754 (1026 to 6479)	25·7% (−67·2 to 467·8)	2211 (1082 to 3631)	−13·7% (−59·4 to 90·1)
Vietnam	2537 (2036 to 3035)	−29·5% (−44·6 to −3·9)	347 567 (117 296 to 547 206)	5·6% (−68·4 to 266·5)	223 067 (134 466 to 338 834)	−22·2% (−55·6 to 38·9)
**Oceania**	**177 (129 to 247)**	**5·6% (−12·0 to 26·8)**	**26 404 (12 740 to 41 462)**	**7·5% (−51·7 to 142·9)**	**20 762 (13 261 to 29 684)**	**5·2% (−31·7 to 68·3)**
American Samoa	2 (1 to 2)	101·3% (45·0 to 157·7)	302 (99 to 451)	25·5% (−58·6 to 306·1)	186 (111 to 277)	46·9% (−20·9 to 204·0)
Federated States of Micronesia	2 (1 to 2)	7·1% (−25·9 to 48·3)	308 (100 to 455)	8·6% (−65·3 to 279·6)	207 (119 to 303)	2·0% (−44·5 to 91·7)
Fiji	21 (15 to 26)	2·8% (−26·8 to 46·8)	2990 (1046 to 4590)	5·5% (−69·5 to 228·8)	2307 (1472 to 3329)	5·1% (−41·4 to 85·0)
Guam	1 (1 to 1)	51·5% (20·8 to 92·8)	516 (166 to 807)	10·3% (−66·6 to 290·6)	234 (105 to 394)	17·6% (−50·1 to 224·1)
Kiribati	4 (3 to 5)	4·1% (−15·9 to 30·0)	342 (94 to 562)	6·1% (−73·0 to 305·4)	382 (262 to 526)	2·5% (−37·7 to 61·3)
Marshall Islands	1 (1 to 1)	14·4% (−13·8 to 45·1)	207 (74 to 322)	9·7% (−66·1 to 284·3)	143 (84 to 220)	7·4% (−43·9 to 96·5)
Northern Mariana Islands	1 (1 to 1)	0·6% (−26·9 to 38·8)	355 (120 to 543)	1·8% (−68·0 to 221·1)	151 (66 to 262)	−7·8% (−64·3 to 127·4)
Papua New Guinea	128 (83 to 192)	2·5% (−19·3 to 31·3)	16 671 (4342 to 30 406)	11·5% (−73·5 to 415·7)	14 271 (7770 to 22 092)	4·9% (−46·0 to 111·0)
Samoa	2 (2 to 3)	−2·6% (−23·7 to 24·1)	586 (197 to 869)	7·7% (−67·0 to 227·1)	318 (168 to 497)	−2·4% (−49·8 to 87·1)
Solomon Islands	11 (7 to 15)	22·5% (−6·7 to 62·6)	1599 (485 to 2499)	14·0% (−63·3 to 321·0)	1249 (723 to 1808)	16·1% (−34·6 to 115·9)
Tonga	1 (1 to 1)	−2·9% (−25·7 to 29·8)	295 (88 to 453)	7·7% (−68·6 to 234·5)	143 (68 to 236)	1·0% (−53·6 to 130·5)
Vanuatu	5 (4 to 7)	22·0% (−3·9 to 58·6)	689 (186 to 1134)	11·8% (−72·8 to 317·5)	550 (330 to 814)	17·2% (−36·7 to 123·7)
**North Africa and Middle East**	**7356 (6392 to 8157)**	**−29·0% (−38·5 to −7·2)**	**2 085 190 (1 585 837 to 2 583 210)**	**0·6% (−25·8 to 35·4)**	**1 112 639 (835 154 to 1 428 785)**	**−22·0% (−39·3 to −0·2)**
Afghanistan	997 (462 to 1585)	−5·0% (−20·6 to 20·1)	81 796 (16 564 to 158 200)	8·6% (−78·5 to 478·8)	95 821 (47 566 to 151 640)	−3·5% (−42·3 to 81·1)
Algeria	440 (348 to 574)	−27·3% (−44·5 to −4·7)	143 986 (52 342 to 210 520)	−0·8% (−64·0 to 192·9)	72 891 (36 536 to 115 429)	−20·3% (−61·6 to 56·4)
Bahrain	19 (15 to 23)	−21·7% (−39·2 to 3·2)	5371 (1861 to 7938)	0·2% (−68·2 to 224·9)	2550 (1356 to 4155)	−19·9% (−63·7 to 73·3)
Egypt	498 (389 to 657)	−30·2% (−44·3 to −8·9)	347 306 (119 883 to 516 249)	22·6% (−62·8 to 261·1)	154 089 (64 781 to 263 047)	−4·2% (−62·7 to 113·6)
Iran	765 (627 to 937)	−29·3% (−46·3 to −3·1)	344 959 (114 307 to 517 959)	18·1% (−64·5 to 290·2)	153 434 (74 033 to 259 394)	−13·6% (−63·1 to 77·5)
Iraq	506 (388 to 632)	−13·6% (−37·5 to 14·7)	148 451 (47 636 to 224 948)	7·8% (−66·0 to 247·4)	82 976 (43 353 to 132 061)	−6·7% (−57·2 to 74·2)
Jordan	62 (48 to 78)	−28·9% (−46·4 to −1·6)	28 226 (10 556 to 40 921)	1·4% (−63·0 to 171·4)	12 215 (6260 to 20 483)	−19·3% (−61·6 to 71·7)
Kuwait	22 (17 to 29)	−16·7% (−37·9 to 8·8)	13 410 (4695 to 20 390)	7·2% (−66·0 to 260·3)	5018 (2149 to 8992)	−10·5% (−65·7 to 119·0)
Lebanon	41 (30 to 56)	−52·1% (−66·7 to −24·3)	18 771 (5963 to 29 876)	0·7% (−71·5 to 284·7)	7294 (3284 to 13 335)	−32·8% (−73·5 to 56·8)
Libya	65 (52 to 78)	−19·1% (−31·5 to −1·2)	17 003 (6200 to 25 165)	1·0% (−65·1 to 222·5)	8743 (4887 to 13 833)	−16·6% (−56·9 to 70·7)
Morocco	923 (471 to 1887)	−18·0% (−40·4 to 16·7)	131 484 (39 352 to 202 772)	−3·2% (−74·9 to 192·2)	91 536 (39 622 to 153 483)	−19·5% (−61·6 to 41·9)
Oman	37 (30 to 45)	−39·8% (−53·6 to −15·7)	16 113 (5483 to 23 524)	0·1% (−67·2 to 210·3)	6815 (3194 to 11 719)	−24·3% (−67·9 to 56·0)
Palestine	99 (87 to 118)	−18·6% (−32·8 to −0·3)	20 243 (7532 to 30 109)	−7·8% (−66·8 to 165·2)	12 442 (7685 to 18 403)	−22·8% (−54·3 to 33·5)
Qatar	11 (8 to 15)	−65·9% (−75·6 to −51·7)	7472 (2440 to 11 236)	−16·1% (−72·7 to 167·5)	2720 (1211 to 4856)	−42·9% (−77·5 to 31·0)
Saudi Arabia	211 (182 to 253)	−35·2% (−47·6 to −11·9)	101 825 (72 566 to 131 166)	9·3% (−23·9 to 62·4)	40 602 (28 238 to 56 390)	−20·9% (−43·3 to 11·1)
Sudan	630 (417 to 899)	−23·5% (−40·7 to 7·9)	100 884 (25 304 to 177 328)	8·9% (−74·7 to 394·6)	78 885 (42 112 to 123 911)	−15·8% (−54·6 to 82·0)
Syria	83 (71 to 95)	−32·0% (−45·1 to −3·3)	63 970 (19 973 to 97 795)	11·6% (−64·7 to 268·7)	24 507 (9115 to 44 697)	−12·8% (−65·3 to 104·3)
Tunisia	112 (81 to 149)	−33·1% (−47·5 to −10·3)	33 543 (11 913 to 49 929)	8·2% (−64·4 to 205·7)	16 040 (8153 to 26 700)	−21·0% (−62·4 to 47·8)
Turkey	1316 (1106 to 1544)	−47·9% (−61·7 to −12·9)	345 186 (134 469 to 493 338)	−18·4% (−70·2 to 127·0)	171 537 (97 074 to 267 928)	−43·5% (−71·0 to 8·2)
United Arab Emirates	115 (82 to 151)	−20·3% (−41·7 to 11·8)	34 858 (12 413 to 52 207)	−1·3% (−67·5 to 228·3)	17 172 (9213 to 27 895)	−19·1% (−64·9 to 82·4)
Yemen	404 (274 to 568)	−26·8% (−42·5 to 6·5)	78 302 (21 865 to 132 676)	7·3% (−73·0 to 426·7)	54 652 (29 282 to 85 361)	−19·6% (−58·3 to 63·3)
**South Asia**	**43 009 (39 131 to 47 849)**	**−33·0% (−42·2 to −15·9)**	**5 272 409 (4 221 404 to 6 351 648)**	**−1·9% (−22·0 to 23·9)**	**3 697 849 (3 076 577 to 4 457 900)**	**−31·7% (−43·1 to −15·6)**
Bangladesh	4449 (3565 to 5648)	−55·3% (−66·6 to −37·3)	530 689 (176 781 to 816 118)	−18·2% (−74·5 to 194·1)	395 800 (265 827 to 558 704)	−48·5% (−68·3 to −17·6)
Bhutan	18 (13 to 24)	−44·3% (−60·2 to −16·7)	2808 (909 to 4471)	−7·1% (−70·5 to 250·4)	1764 (1061 to 2612)	−40·2% (−66·3 to 2·9)
India	34 253 (31 149 to 37 749)	−31·4% (−40·7 to −13·5)	3 934 737 (3 176 019 to 4 766 534)	−1·9% (−20·1 to 21·4)	2 806 946 (2 338 317 to 3 399 136)	−31·7% (−43·0 to −14·9)
Nepal	709 (565 to 896)	−31·6% (−50·0 to −6·7)	113 530 (35 311 to 174 642)	11·7% (−65·0 to 341·8)	68 811 (42 894 to 102 692)	−28·2% (−59·6 to 24·0)
Pakistan	3580 (2853 to 4286)	−8·9% (−27·8 to 13·4)	690 645 (204 907 to 1 169 829)	9·5% (−69·4 to 412·5)	424 529 (249 368 to 664 059)	−5·7% (−49·7 to 92·7)
**Southern sub-Saharan Africa**	**5693 (5216 to 6245)**	**−5·1% (−18·2 to 9·1)**	**383 628 (280 596 to 496 987)**	**1·4% (−30·4 to 45·5)**	**389 805 (331 721 to 457 948)**	**−8·0% (−21·3 to 7·6)**
Botswana	171 (76 to 271)	1·8% (−48·5 to 65·0)	13 791 (4748 to 21 418)	13·7% (−63·1 to 317·0)	12 454 (7049 to 18 844)	1·5% (−41·2 to 67·0)
Lesotho	199 (143 to 261)	30·6% (−21·2 to 90·3)	7471 (1509 to 14 829)	30·5% (−81·0 to 734·2)	12 171 (8310 to 16 454)	33·3% (−23·9 to 118·8)
Namibia	159 (101 to 234)	−20·9% (−44·3 to 5·8)	11 753 (3202 to 20 350)	−2·9% (−73·6 to 308·6)	11 283 (6576 to 17 192)	−20·1% (−49·7 to 27·1)
South Africa	4429 (4005 to 4946)	−12·1% (−25·4 to 2·6)	294 658 (203 182 to 388 082)	0·6% (−34·5 to 54·8)	296 686 (248 902 to 352 893)	−13·3% (−28·3 to 3·7)
Swaziland	100 (67 to 138)	5·2% (−27·0 to 43·9)	6593 (1618 to 11 383)	9·8% (−71·4 to 356·8)	7357 (4722 to 10 646)	8·0% (−31·1 to 60·3)
Zimbabwe	635 (509 to 780)	81·5% (34·3 to 206·5)	49 362 (12 760 to 87 483)	4·7% (−75·1 to 314·2)	49 854 (34 019 to 70 851)	49·3% (−14·1 to 165·5)
**Western sub-Saharan Africa**	**8732 (6967 to 11 218)**	**7·3% (−7·6 to 24·3)**	**1 376 947 (760 842 to 2 178 156)**	**7·1% (−43·9 to 123·2)**	**1 010 670 (715 404 to 1 435 946)**	**−1·2% (−30·4 to 44·9)**
Benin	274 (229 to 327)	42·0% (17·1 to 73·8)	51 645 (11 420 to 99 962)	13·0% (−75·5 to 512·1)	34 894 (18 694 to 59 815)	15·1% (−47·3 to 157·1)
Burkina Faso	395 (328 to 480)	−7·7% (−25·2 to 16·6)	49 576 (7985 to 120 431)	1·4% (−84·7 to 617·9)	44 226 (25 505 to 74 362)	−8·2% (−54·4 to 96·0)
Cameroon	663 (467 to 897)	63·2% (24·4 to 105·8)	107 383 (20 059 to 229 080)	22·6% (−80·9 to 661·1)	77 901 (40 785 to 136 899)	31·8% (−40·1 to 213·4)
Cape Verde	17 (14 to 22)	−4·0% (−28·7 to 29·4)	3383 (911 to 5671)	15·8% (−72·4 to 389·1)	1882 (1033 to 3162)	−2·5% (−51·1 to 92·8)
Chad	357 (274 to 435)	48·8% (22·6 to 82·5)	44 949 (7136 to 105 860)	24·8% (−83·8 to 805·7)	39 677 (22 087 to 68 741)	31·4% (−39·9 to 195·5)
Côte d'Ivoire	693 (535 to 855)	60·0% (31·0 to 93·5)	81 378 (18 215 to 168 075)	18·7% (−76·2 to 478·1)	68 594 (41 257 to 110 732)	29·9% (−34·5 to 164·5)
The Gambia	38 (30 to 47)	39·8% (15·1 to 68·7)	8257 (2371 to 13 718)	11·6% (−69·4 to 334·0)	5113 (2874 to 8091)	10·5% (−43·6 to 131·6)
Ghana	411 (342 to 491)	46·2% (17·0 to 88·0)	137 757 (34 782 to 240 697)	25·9% (−71·4 to 439·9)	70 879 (30 793 to 125 142)	17·6% (−53·6 to 214·1)
Guinea	321 (264 to 382)	45·4% (17·4 to 79·4)	40 317 (7713 to 89 091)	8·7% (−81·2 to 651·8)	33 725 (19 721 to 57 367)	14·3% (−43·6 to 149·4)
Guinea-Bissau	64 (48 to 78)	36·4% (9·4 to 71·2)	4710 (762 to 12 028)	15·1% (−84·8 to 848·2)	5344 (3332 to 8534)	18·0% (−39·0 to 144·5)
Liberia	95 (78 to 116)	34·2% (10·5 to 64·7)	20 420 (4697 to 41 125)	1·1% (−81·3 to 366·6)	12 594 (6363 to 22 215)	−0·8% (−58·4 to 133·0)
Mali	377 (284 to 473)	14·5% (−16·4 to 58·1)	41 309 (5316 to 98 257)	28·9% (−82·0 to 861·8)	37 675 (21 041 to 62 799)	7·0% (−48·0 to 106·9)
Mauritania	73 (48 to 105)	14·0% (−20·4 to 64·7)	22 889 (6600 to 37 382)	8·7% (−72·5 to 300·4)	12 189 (5764 to 20 521)	0·8% (−55·5 to 134·5)
Niger	451 (352 to 551)	28·3% (−4·4 to 79·2)	59 803 (11 391 to 127 616)	12·6% (−81·0 to 599·0)	49 820 (27 853 to 82 354)	6·7% (−48·8 to 119·6)
Nigeria	3772 (2141 to 6507)	−17·0% (−42·6 to 13·9)	577 948 (92 319 to 1 265 749)	−2·8% (−85·1 to 499·3)	428 168 (193 932 to 775 350)	−17·2% (−65·2 to 73·0)
São Tomé and Príncipe	3 (2 to 4)	25·2% (−5·2 to 59·3)	1050 (324 to 1684)	17·0% (−67·6 to 374·3)	568 (272 to 935)	8·7% (−50·9 to 147·3)
Senegal	378 (327 to 435)	45·1% (23·7 to 68·7)	74 912 (19 138 to 127 400)	10·4% (−72·3 to 296·9)	49 079 (27 383 to 77 078)	13·1% (−43·3 to 130·2)
Sierra Leone	162 (122 to 200)	38·6% (16·3 to 67·4)	18 035 (2960 to 42 479)	13·7% (−85·1 to 776·9)	16 196 (9012 to 28 000)	15·5% (−45·2 to 138·6)
Togo	188 (156 to 221)	46·1% (19·8 to 72·7)	31 210 (6230 to 61 789)	−5·3% (−81·8 to 435·8)	22 142 (11 885 to 36 811)	6·0% (−52·7 to 154·1)
**Eastern sub-Saharan Africa**	**8580 (7777 to 9459)**	**−24·7% (−37·1 to −2·9)**	**1 520 535 (1 028 359 to 2 024 454)**	**2·5% (−32·9 to 55·0)**	**1 058 588 (823 366 to 1 353 425)**	**−17·6% (−36·3 to 9·3)**
Burundi	279 (225 to 342)	−26·0% (−44·5 to 11·7)	41 575 (8444 to 79 189)	−5·5% (−83·7 to 405·0)	32 995 (18 709 to 53 315)	−21·6% (−60·2 to 58·3)
Comoros	18 (14 to 22)	−22·7% (−41·4 to 3·8)	3500 (1038 to 5908)	−7·3% (−76·4 to 254·9)	2307 (1302 to 3569)	−21·3% (−60·0 to 51·1)
Djibouti	24 (18 to 32)	−9·2% (−34·9 to 20·5)	4357 (1068 to 7626)	1·9% (−75·7 to 374·4)	2979 (1600 to 4730)	−8·9% (−55·4 to 84·2)
Eritrea	134 (105 to 162)	−21·8% (−41·8 to 7·6)	20 890 (4835 to 37 975)	2·4% (−77·7 to 364·6)	15 943 (8712 to 25 452)	−14·8% (−56·7 to 60·8)
Ethiopia	2671 (2124 to 3239)	−42·3% (−59·0 to −12·7)	371 758 (106 621 to 596 895)	−5·8% (−75·4 to 300·5)	286 989 (175 383 to 427 015)	−35·3% (−65·0 to 20·3)
Kenya	554 (434 to 680)	−1·6% (−17·4 to 17·4)	200 780 (160 992 to 244 896)	4·1% (−12·1 to 26·9)	102 527 (76 594 to 132 453)	−3·5% (−17·6 to 12·4)
Madagascar	560 (430 to 711)	−13·3% (−32·9 to 14·5)	79 682 (17 696 to 150 960)	−4·3% (−81·3 to 392·8)	62 449 (36 885 to 100 163)	−14·3% (−56·8 to 67·4)
Malawi	431 (322 to 551)	−9·7% (−35·3 to 25·9)	55 657 (11 206 to 114 932)	2·2% (−83·0 to 473·1)	45 071 (26 037 to 72 907)	−11·0% (−55·8 to 77·1)
Mozambique	508 (386 to 691)	−22·9% (−42·1 to 4·6)	112 389 (19 239 to 231 743)	16·1% (−79·8 to 499·2)	73 287 (34 697 to 129 718)	−9·4% (−61·6 to 104·0)
Rwanda	246 (201 to 301)	−26·1% (−48·0 to 19·5)	51 061 (12 640 to 87 890)	8·5% (−74·4 to 413·2)	32 509 (17 651 to 51 320)	−18·2% (−60·4 to 80·0)
Somalia	304 (224 to 384)	−10·4% (−29·6 to 15·3)	28 138 (4753 to 60 869)	4·5% (−84·1 to 694·3)	28 857 (16 599 to 45 466)	−8·5% (−51·5 to 79·8)
South Sudan	298 (224 to 386)	0·4% (−23·3 to 33·8)	35 504 (6002 to 80 778)	3·3% (−84·5 to 623·6)	31 096 (17 753 to 53 863)	−3·0% (−52·8 to 110·0)
Tanzania	1162 (966 to 1393)	−18·5% (−35·2 to 3·9)	246 202 (54 678 to 428 813)	6·2% (−75·5 to 380·8)	155 696 (85 488 to 249 855)	−10·8% (−56·1 to 82·6)
Uganda	936 (783 to 1112)	−11·5% (−29·3 to 19·2)	194 280 (41 805 to 383 272)	5·1% (−80·2 to 434·2)	130 523 (67 857 to 222 380)	−5·2% (−55·9 to 119·1)
Zambia	455 (316 to 621)	24·2% (−15·6 to 67·9)	73 796 (15 706 to 137 808)	13·0% (−76·4 to 521·1)	54 989 (29 577 to 89 152)	14·5% (−44·9 to 143·7)
**Central sub-Saharan Africa**	**2393 (1871 to 3011)**	**−13·8% (−30·3 to 12·8)**	**272 787 (113 188 to 480 953)**	**3·7% (−59·6 to 162·7)**	**253 362 (171 108 to 371 268)**	**−11·5% (−41·2 to 42·1)**
Angola	530 (384 to 706)	−18·9% (−47·3 to 36·2)	71 859 (13 396 to 143 895)	6·6% (−80·3 to 474·3)	61 288 (35 567 to 96 825)	−15·6% (−57·1 to 74·7)
Central African Republic	149 (90 to 219)	−0·5% (−20·8 to 25·5)	7078 (1005 to 18 481)	−0·7% (−86·4 to 827·7)	11 901 (6419 to 19 191)	−2·1% (−40·9 to 65·6)
Congo (Brazzaville)	97 (70 to 126)	−24·7% (−45·7 to 13·0)	14 479 (3508 to 26 699)	−5·3% (−79·0 to 341·4)	11 085 (6355 to 17 066)	−20·4% (−57·8 to 65·6)
Democratic Republic of the Congo	1573 (1186 to 2099)	−11·1% (−30·4 to 13·5)	170 264 (31 635 to 351 352)	3·5% (−79·9 to 522·2)	163 335 (98 089 to 259 549)	−9·2% (−50·2 to 74·1)
Equatorial Guinea	11 (7 to 17)	−56·9% (−77·8 to −4·5)	2558 (528 to 5136)	30·6% (−75·4 to 554·1)	1514 (694 to 2629)	−36·3% (−74·1 to 55·5)
Gabon	34 (26 to 45)	−16·4% (−40·1 to 19·8)	6549 (1594 to 11 829)	−0·9% (−75·6 to 267·2)	4238 (2322 to 6845)	−14·3% (−58·7 to 69·5)

95% uncertainty intervals are in parentheses. DALYs=disability-adjusted life-years. SDI=Socio-demographic Index.

Globally, in 2016, there were 1·4 million (95% UI 1·2–1·6) idiopathic epilepsy cases in men and 1·3 million (1·1–1·6) cases in women, with age-standardised incidence rates of 38·9 per 100 000 person-years (32·7–45·7) for men and 37·1 per 100 000 person-years (30·8–44·1) for women. Between 1990 and 2016, there were no significant changes in both age-standardised incidence rates (35·8 per 100 000 person-years [30·1–42·0] in 1990 and 38·0 per 100 000 person-years [31·7–45·1] in 2016) and absolute number of people (2·1 million [1·7–2·4] in 1990 and 2·8 million [2·3–3·3] in 2016) with incident idiopathic epilepsy. There were four times geographical variations in the age-standardised incidence rates of idiopathic epilepsy, with the highest rates observed in Ecuador (70·9 per 100 000 person-years [22·3–112·5]) and Mexico (56·0 per 100 000 person-years [41·0–72·0]) and the lowest rates in North Korea (17·0 per 100 000 person-years [5·7–28·2]) and China (19·7 per 100 000 person-years [14·2–25·6]).

**Figure 1:**
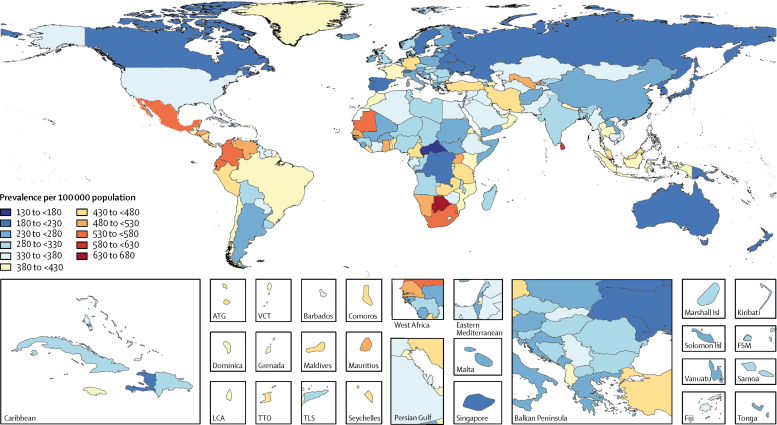
Age-standardised prevalence per 100 000 of idiopathic epilepsy for both sexes, 2016 ATG=Antigua and Barbuda. FSM=Federated States of Micronesia. Isl=Islands. LCA=Saint Lucia. TLS=Timor-Leste. TTO=Trinidad and Tobago. VCT=Saint Vincent and the Grenadines.

**Figure 2:**
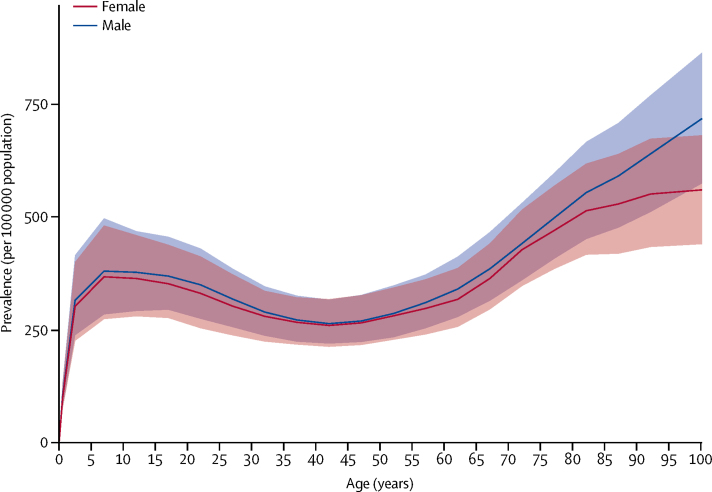
Global prevalence of idiopathic epilepsy by age and sex, 2016 Shaded areas show 95% uncertainty intervals.

In 2016, the global age-standardised prevalence of all active epilepsy (idiopathic and secondary) was 621·5 per 100 000 population (95% UI 540·1–737·0). It varied from a low of 311·0 per 100 000 population (253·4–370·5) in Japan to a high of 1287·7 per 100 000 population (754·4–1791·3) in Cape Verde. The prevalence of idiopathic epilepsy was 326·7 per 100 000 population (278·4–378·1). The prevalence was 329·3 per 100 000 population (280·3–381·2) in men and 318·9 per 100 000 population (271·1–369·4) in women. Highest prevalence was found in eastern, western, and southern sub-Saharan Africa regions, central Asia, central and Andean Latin America, and southeast Asia (figure 1). Prevalence increased with age, with peaks at ages 5–9 years (374·8 [280·1–490·0]) and at older than 80 years (545·1 [444·2–652·0]). Prevalence was similar in men and women (figure 2).

**Figure 3:**
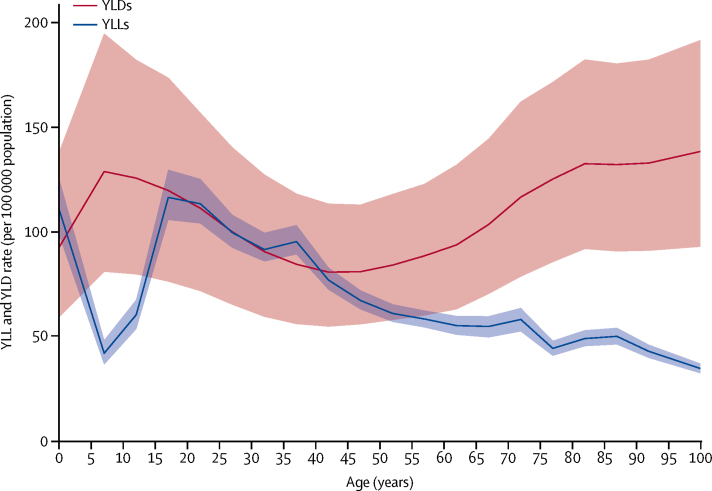
Global years lived with disability (YLDs) and years of life lost (YLLs) rates due to idiopathic epilepsy by age, 2016 Shaded areas show 95% uncertainty intervals.

Global age-standardised DALY rates of idiopathic epilepsy were 182·6 per 100 000 population (95% UI 149·0–223·5; 163·6 per 100 000 population [130·6–204·3] for women and 201·2 per 100 000 population [166·9–241·4] for men). The higher DALY rates in men were due to a higher YLL rate of 96·1 per 100 000 population (89·4–104·2) in men than in women (63·5 per 100 000 population [56·6–70·1]). Global age-standardised YLD rates in 2016 were similar between men (105·1 per 100 000 population [70·8–145·5]) and women (100·1 per 100 000 population [68·0–138·6]). YLLs peaked at age under 5 years and at ages of 15–19 years and then decreased progressively with age (figure 3). YLDs peaked at 5–9 years of age, decreased until 40–49 years, and increased progressively to the oldest age group.

Between 1990 and 2016, age-standardised prevalence of idiopathic epilepsy did not change (6·0% [95% UI −4·0 to 16·7]), but a significant decrease was found in age-standardised mortality rates (24·5% [10·8 to 31·8]) and in age-standardised DALY rates (19·4% [9·0 to 27·6]). For further details on prevalence, incidence, deaths, DALYs, YLLs, and YLDs for both sexes, all ages, countries, and territories, please refer to the GBD Results Tool.

Counts for prevalent cases, deaths, and DALYs, and the corresponding percentage change in age-standardised rates between 1990 and 2016 of idiopathic epilepsy, varied across countries (table 1). There was a significant difference in age-standardised prevalence of idiopathic epilepsy in 2016, ranging from 648·9 per 100 000 population (95% UI 222·8–1012·3) in Botswana to 139·7 per 100 000 population (20·1–366·9) in the Central African Republic. The wide UIs, in part, reflect the heterogeneity of data sources for the estimates of all epilepsy. However, the largest source of uncertainty comes from modelling the sparse data on the proportion of all epilepsy that is idiopathic or secondary to other causes. No region, or even country, had a significant change in age-standardised prevalence between 1990 and 2016. Age-standardised mortality rates of idiopathic epilepsy decreased significantly in 74 countries, mostly from western and central Europe, Australasia, high-income North America, high-income Asia Pacific, Latin America, the Caribbean, and north Africa and the Middle East. By contrast, mortality rates increased in 22 countries, predominantly from western sub-Saharan Africa, and occasionally also from Europe (Belgium, Denmark, Germany, Iceland, and Poland) and southeast Asia (Thailand). As a consequence of changes in mortality rates and subsequently YLL rates of idiopathic epilepsy, age-standardised DALY rates decreased significantly in central Latin America, east Asia, south Asia, and north Africa and the Middle East (table 1). However, in individual countries, a significant decrease was found only in the UK, Mexico, China, Bangladesh, and India.

**Figure 4:**
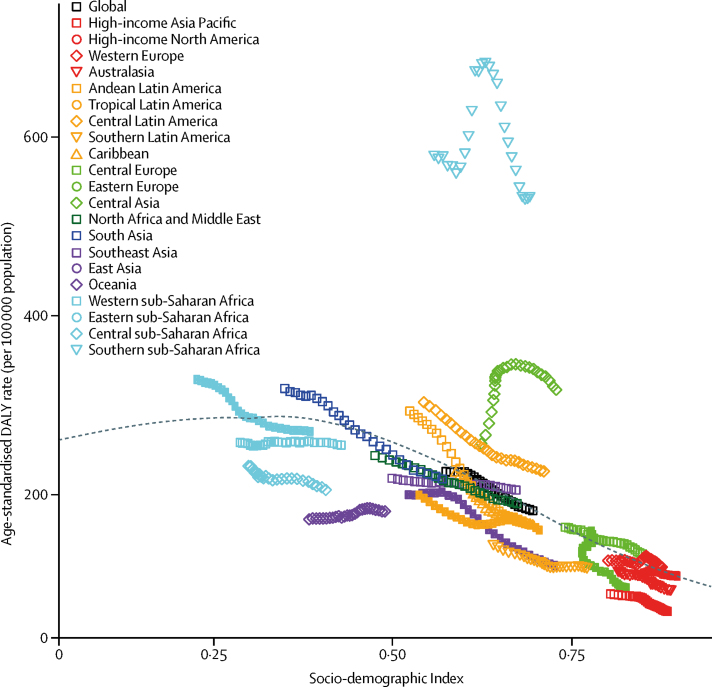
Age-standardised disability-adjusted life-year (DALY) rates for idiopathic epilepsy in 21 GBD regions by Socio-demographic Index, 1990–2016 Age-standardised DALY rates are plotted for 21 world regions between 1990 and 2016 against their Socio-demographic Index values. Points from left to right represent the values from 1990 to 2016. The black line represents the predicted values based on a regression of all country values for all years.

Because of the wide UIs around prevalence, no country experienced a significant change between 1990 and 2016 (table 1). By contrast, the reduction of age-standardised deaths varied by SDI quintiles, with large reductions in the three middle SDI quintiles, but no significant change in the low and high SDI quintiles. Expected values of age-standardised DALY rates decreased from an SDI value of 0·3 upward (figure 4). Most regions saw a steady decrease over time, with values close to the expected line. Rates in Oceania and western sub-Saharan Africa remained mostly unchanged over the estimation period. Rates in central Asia and southern sub-Saharan Africa rose to a peak halfway through the estimation period and then declined.

About two-thirds of the gap in age-standardised DALY rates between low SDI quintile countries (249·2 per 100 000 population [95% UI 202·3–307·6]) and high SDI quintile countries (110·6 per 100 000 population [84·2–143·7]) were due to a difference in YLL rates (126·92 per 100 000 population [114·81–140·13] for low SDI countries and 34·05 per 100 000 population [32·48–36·28] for high SDI countries), and one-third was due to lesser severity of disease as measured in YLDs. Prevalence of idiopathic epilepsy was similar among the quintiles of SDI. In 2016, there was a strong gradient in the prevalence of idiopathic epilepsy by severity. Low SDI quintile countries had the highest age-standardised prevalence of severe epilepsy and the lowest age-standardised prevalence of treated epilepsy without seizures, with the opposite being the case in high SDI countries (table 2).

**Table 2:** Age-standardised prevalence per 100 000 population and YLDs of idiopathic epilepsy in 2016, and its sequelae globally and by SDI quintiles

	**Idiopathic epilepsy**		**Seizure-free, treated idiopathic epilepsy**		**Less severe idiopathic epilepsy**		**Severe epilepsy**	
	Prevalence	YLDs	Prevalence	YLDs	Prevalence	YLDs	Prevalence	YLDs
Global	326·7 (278·4–378·1)	102·6 (69·5–142·0)	65·9 (50·6–83·0)	3·1 (1·8–4·8)	125·5 (98·3–155·1)	30·7 (19·4–45·0)	135·3 (103·3–170·8)	68·7 (45·0–97·8)
High SDI	309·2 (247·4–372·3)	76·5 (50·3–109·7)	137·9 (99·7–177·8)	6·5 (3·6–10·0)	63·3 (44·9–87·2)	15·4 (9·3–23·6)	108·0 (72·7–153·8)	54·6 (31·7–84·4)
High-middle SDI	295·6 (231·4–359·5)	86·7 (56·0–124·8)	83·4 (57·0–112·5)	3·9 (2·2–6·3)	95·4 (65·3–132·7)	23·4 (13·7–36·5)	116·8 (75·2–166·6)	59·3 (34·3–91·1)
Middle SDI	348·3 (294·6–409·9)	109·4 (73·8–150·4)	65·5 (46·4–87·4)	3·1 (1·7–5·0)	143·1 (109·5–184·6)	35·1 (21·8–51·9)	139·6 (102·9–182·0)	71·2 (44·2–102·5)
Low-middle SDI	326·6 (256·9–400·7)	108·3 (69·8–154·6)	34·5 (22·5–49·0)	1·6 (0·9–2·8)	153·9 (110·1–200·8)	37·3 (22·3–56·8)	138·2 (94·6–190·0)	69·5 (41·4–105·9)
Low SDI	325·3 (242·3–416·4)	122·5 (76·8–178·0)	15·1 (7·4–25·8)	0·7 (0·3–1·4)	131·0 (83·7–191·7)	31·8 (17·6–53·7)	179·3 (123·5–250·2)	90·0 (53·5–138·3)

95% uncertainty intervals are in parentheses. Severe epilepsy is defined as more than one seizure per month; less severe epilepsy is defined as those who have 1–11 seizures per year or did not have a seizure in the past year while untreated but still fulfilling criteria of active epilepsy. SDI=Socio-demographic Index. YLDs=years lived with disability.

The only risk quantified in GBD for idiopathic epilepsy was alcohol use, estimated to be responsible for 18·9% (95% UI 14·6–23·1) of global DALYs from epilepsy in men (7 524 103·60 [6 254 535·71–9 024 594·20]) and 8·2% (5·8–10·5) in women (5 968 147·15 [4 760 417·45–7 452 692·07]).

## Discussion

Between 1990 and 2016, a significant reduction was observed in the mortality rate in people with idiopathic epilepsy and, to a lesser extent, a reduction was found in DALY rates, a comprehensive measure of the burden of the disease, when adjusted for age. This finding probably reflects improvements in access to treatment leading to a lower risk of death and lesser severity of the disease. Nevertheless, a substantial treatment gap remains (due to insufficient financial resources, misconceptions, and stigma^22^) that can explain the larger proportion of severe epilepsies and higher case fatality when comparing high SDI with middle and low SDI countries.

Our estimate of 45·9 million cases of idiopathic active and secondary epilepsy in 2016 is higher than the 32·7 million cases reported from a meta-analysis of 65 prevalence studies,^7^ but it seems that the estimate of the number of cases of active epilepsy in rural populations in low-income countries was reported as 17 million in the meta-analysis, whereas our interpretation of the results is that it should have been 37 million. Furthermore, it is not clearly stated for which year the estimate was made in the meta-analysis. However, similar to findings from that meta-analysis, large variations were observed in the prevalence of epilepsy in this study, but there was no clear pattern by development status or by location. This finding raises the question of how much of the variation we estimated between countries is real or an artifact of measurement error we have been unable to control for. The unknown cause of epilepsy, apart from an association with alcohol use, makes it difficult to fit estimates to sparse and heterogeneous data.

In 2016, epilepsy accounted for more than 13 million DALYs, that is 0·56% of total DALYs globally. The numbers are significantly higher than the projected estimates from WHO (7·4 million),^5^ but the proportion of total DALYs attributable to epilepsy in the WHO report (0·50%) was almost identical despite multiple measurement differences making results incomparable. In that report, epilepsy was defined in accordance with the 2005 ILAE and International Bureau for Epilepsy definition as a disorder of the brain characterised by an enduring predisposition to generate epileptic seizures.^23^ This definition is in keeping with the definition used in the present study. Other population-based studies addressed the burden of epilepsy using the DALY metrics. These studies were done in China,^24^ India,^25^ and South Africa.^26^ However, the results of these studies cannot be compared with ours because of the different methodology and the regional perspective.

Apart from the major changes in how DALYs are defined since GBD 2010 (no more discounting or age-weighting, a prevalence instead of incidence approach to measuring non-fatal outcomes, and disability weights derived from large population surveys rather than a small panel of health experts), the largest difference is that in the past decade, we have developed statistical models that can evaluate all available epidemiological evidence rather than relying on an analyst to determine a single data source to describe prevalence or incidence in a country.

Variables such as race or ethnicity and socioeconomic level might be also inter-related. Our and others' findings^6^ support the concept that epilepsy and poverty might have a bidirectional association. The inverse association between the burden of epilepsy and sociodemographic status is in line with other neurological disorders^8^ and with published reports from low-income countries and from people with low incomes in high-income countries.^27^, ^28^, ^29^ Inequalities in health might also vary among members of the same population.^30^ Low socioeconomic status is also associated with risk factors for epilepsy.^31^ We should, however, argue that if we find an important link between the development status of countries and epilepsy outcomes, it is likely that much larger variation in outcomes exists at the individual level depending on a person's socioeconomic attributes.

The major strength of this study is the worldwide assessment of the burden of all major diseases, including epilepsy, using the same methodology and modelling measures. Another strength is the continuous refinement of the available data through input from new original sources and the use of more sophisticated statistical methods as these develop. There are, however, some general and disease-specific limitations. First, as original epidemiological data were not available for all countries, Bayesian statistical models were used to estimate deaths and disease prevalence for countries with missing information. The inclusion of data sources from new original studies in countries for which no data were available in a previous iteration of GBD can lead to more precise estimates that might vary considerably from previous predicted values. The annual updates of GBD provide an opportunity to improve on estimates as new data or new methods become available. Second, the disability weights used for the calculation of YLDs might not be uniform across populations and sociodemographic levels. However, population surveys in nine countries did not find systematic variation in disability weights across populations or within the same population as a function of education.^17^, ^32^, ^33^ Third, the 95% UI used to define the precision of the estimates are wide, reflecting the overall uncertainty of the estimates and, as a consequence, limiting the ability to find differences across countries. This finding can explain why few countries showed a significant change in DALY rates. The main source of uncertainty around the incidence and prevalence estimates of idiopathic epilepsy comes from the sparse and heterogeneous data about the proportion of people with idiopathic as opposed to secondary epilepsy and, for idiopathic epilepsy, on the distribution of (presumed) genetic and cryptogenic forms. Likewise, deaths as part of idiopathic epilepsy might include deaths of people with secondary epilepsy. Further uncertainty comes from our definition of severity and the estimates of the proportion of people with severe epilepsy, people with less severe epilepsy, and those with no seizures while on treatment. A third source of uncertainty for the YLD estimates comes from the wide uncertainty bounds around the disability weights. New data collection on these various proportions that determine the sequelae of epilepsy would have the greatest bearing on reducing UIs. Fourth, epilepsy is correlated with somatic and psychiatric comorbidities^34^ and injuries,^35^ as well as a host of diseases associated with stigma and poverty.^36^ Here, the correction for comorbidity was based on the assumption that diseases and their sequelae are independent. Future improvements of the GBD modelling should include dependent comorbidity. Fifth, the use of medical claims data could introduce a systematic bias as people who are not under treatment or are excluded from health insurance would not be counted. For the 3 years of claims data from the USA, we applied a correction based on a comparison with representative survey data. In coming years, we hope to include claims data from other countries and more comprehensive claims data from the USA that are less biased towards individuals with private health insurance only. The challenge will be to find representative survey data in those countries to make credible adjustments. Sixth, the peak in DALY rates in southern sub-Saharan Africa coincides with the peak in deaths as a result of HIV or AIDS in 2005 and suggests that despite an effort to correct deaths miscoded to non-HIV causes in the South African vital registration data, we might have left some remaning deaths that should have been reassigned to HIV or AIDS. However, even if the peak is an artifact, these regions still have much higher than expected DALY rates for idiopathic epilepsy. The reason for the recorded increase and then decrease in deaths in vital registration data from central Asia is less clear. Seventh, the higher mortality rate of idiopathic epilepsy in some western European countries than in low-income and middle-income countries cannot be easily explained and might reflect differences in cause of death certification practices. Last, epilepsy in this report is defined by having at least two unprovoked seizures and is in contrast with the recent ILAE definition that includes a single unprovoked seizure judged at high risk of relapse.^37^ However, almost all published reports included patients with recurrent seizures and perhaps the inclusion of individuals with single seizures would increase the burden of the disease.

In conclusion, our findings have important implications for health service planning. The decrease in death and DALY rates in patients with epilepsy between 1990 and 2016 is encouraging, but the changes varied across geographical areas and, where data were available, within countries. Furthermore, changes were linked to the sociodemographic development status, which should prompt more action in economically deprived areas. The success of reducing the burden of idiopathic epilepsy relies mostly on access to treatment. Health service planners and providers also need to be aware that patients with epilepsy are more often poor and marginalised because of stigma, requiring a greater effort to reach them than might be the case for most other diseases.
