# Correction to *Lancet Neurol* 2019; 18: 357–75

**DOI:** 10.1016/S1474-4422(19)30120-6

**Published:** 2019-05

*GBD 2016 Epilepsy Collaborators. Global, regional, and national burden of epilepsy, 1990–2016: a systematic analysis for the Global Burden of Disease Study 2016.* Lancet Neurol *2019; **18:** 357–75—*In the author list, Emma Nichols should have been listed after Giorgia Giussani. This correction has been made to the online version as of April 10, 2019.

