# Supplementary material for: Global, regional, and national burden of epilepsy, 1990–2016: a systematic analysis for the Global Burden of Disease Study 2016
Source: Lancet Neurol. 2019 Apr;18(4):357–75. doi: 10.1016/S1474-4422(18)30454-X (PMC6416168; doi:10.1016/S1474-4422(18)30454-X)
Supplement: Supplementary appendix [file mmc1.pdf]

THE LANCET
Neurology

Supplementary appendix

This appendix formed part of the original submission and has been peer reviewed. We post it as supplied by the authors.

Supplement to: GBD 2016 Epilepsy Collaborators. Global, regional, and national burden of epilepsy, 1990–2016: a systematic analysis for the Global Burden of Disease Study 2016. *Lancet Neurol* 2019; published online Feb 14. http://dx.doi.org/10.1016/S1474-4422(18)30454-X.

Appendix

Summary of General Global Burden of Disease Study Methods

The Institute for Health Metrics and Evaluation with a growing collaboration of scientists produces annual updates of the Global Burden of Disease study. Estimates span the period from 1990 to the most recent completed year. By the time of the release of GBD 2016 in September 2017, there were over 2,700 collaborators in 132 countries who contributed to this global public good. Annual updates allow incorporation of new data and method improvements to ensure that the most up-to-date information is available to policy makers in a timely fashion to help make resource allocation decisions. In this analysis, we have aggregated results from GBD 2016 for 15 disease and injury outcomes that are generally cared for by neurological services. These include infectious conditions (tetanus, meningitis, encephalitis), stroke, brain and other nervous system cancers, traumatic brain injury, and spinal cord lesion which are classified outside the more narrowly defined category of neurological disorders in GBD (ie, Alzheimer's disease and other dementias, Parkinson's disease, multiple sclerosis, motor neuron disease, idiopathic epilepsy, migraine, tension-type headache, and a rest category of less common other neurological disorders). Compared to a previous analysis based on GBD 2015,[1] we were able to add the non-fatal outcomes of traumatic brain injury and spinal cord lesion, and medication overuse headache is no longer included as a separate cause but quantified as a consequence of the underlying headache types.

In the methods section of this overview paper we present a summary of the general methods of the global burden of disease. In the accompanying disease-specific papers we concentrate on methods that are specific to each disorder. The guiding principle of GBD is to assess health loss due to mortality and disability comprehensively, where we define disability as any departure from full health. In GBD 2016, estimates were made for 195 countries and territories, and 579 subnational locations, for 27 years starting from 1990, for 23 age groups and both sexes. Deaths were estimated for 264 diseases and injuries, while prevalence and incidence were estimated for 328 diseases and injuries. In order to allow meaningful comparisons between deaths and non-fatal disease outcomes as well as between diseases, the data on deaths and prevalence are summarised in a single indicator, the disability-adjusted life-year (DALY). DALYs are the sum of years of life lost (YLLs) and years lived with disability (YLDs). YLLs are estimated as the multiplication of counts of death and a standard, "ideal", remaining life expectancy at the age of death. The standard life expectancy is derived from the lowest observed mortality rates in any population in the world greater than 5 million.[2] YLDs are estimated as the product of prevalence of individual consequences of disease (or "sequelae") times a disability weight that quantifies the relative severity of a sequela as a number between zero (representing "full health") and 1 (representing death). Disability weights have been estimated in nine population surveys and an open-access internet survey in which respondents are asked to choose the "healthier"[3] between random pairs of health states that are presented with a short description of the main features.

All-cause mortality rates are estimated from vital registration data in countries with complete coverage. For other countries, the probabilities of death before age 5 and between ages 15 and 60 are estimated from censuses and surveys asking mothers to provide a history of children ever born and those still alive, and surveys asking adults about siblings who are alive or have passed away. Using model life tables, these probabilities of death are transformed into age-specific death rates by location, year, and sex. GBD has collated a large database of cause of death data from vital registrations and verbal autopsy surveys in which relatives are asked a standard set of questions to ascertain the likely cause of death, supplemented with police and mortuary data for injury deaths in countries with no other data. For countries with vital registration data, the completeness is assessed with demographic methods based on comparing recorded deaths with population counts between two successive censuses. The cause of death information is provided in a large number of different classification systems based on versions of the International

Classification of Diseases or bespoke classifications in some countries. All data are mapped into the disease and injury categories of GBD. All classification systems contain codes that are less informative because they lack a specific diagnosis (eg, unspecified cancer) or refer to codes that cannot be underlying cause of death (eg, low back pain or senility) or are intermediate causes (eg, heart failure or sepsis). Such deaths are redistributed to more precise underlying causes of death.[4] After these redistributions and corrections for under-registration, the data are analysed in CODEm (cause of death ensemble model), a highly systematised tool that runs many different models on the same data and chooses an ensemble of models that best reflects all the available input data. Models are chosen with variations in the statistical approach ("mixed effects" of spatiotemporal Gaussian Process Regression), in the unit of analysis (rates or cause fractions), and the choice of predictive covariates. The statistical performance of all models is tested by holding out 30% of the data and checking how well a model covers the data that were held out. To enforce consistency from CODEm, the sum of all cause-specific mortality rates is scaled to that of the all-cause mortality rates in each age, sex, location, and year category.

Non-fatal estimates are based on systematic reviews of published papers and unpublished documents, survey microdata, administrative records of health encounters, registries, and disease surveillance systems. Our Global Health Data Exchange (GHDx, http://ghdx.healthdata.org/) is the largest repository of health data globally. We first set a reference case definition and/or study method that best quantifies each disease or injury or consequence thereof. If there is evidence of a systematic bias in data that used different case definitions or methods compared to reference data we adjust those data points to reflect what its value would have been if measured as the reference. This is a necessary step if one wants to use all data pertaining to a particular quantity of interest rather than choosing a small subset of data of the highest quality only. DisMod-MR 2.1, a Bayesian meta-regression tool, is our main method of analyzing non-fatal data. It is designed as a geographical cascade where a first model is run on all the world's data, which produces an initial global fit and estimates coefficients for predictor variables and the adjustments for alternative study characteristics. The global fit adjusted by the values of random effects for each of seven GBD super-regions, the coefficients on sex and country predictors, are passed down as data to a model for each super-region together with the input data for that geography. The same steps are repeated going from super-region to 21 region fits and then to 195 fits by country and where applicable a further level down to subnational units. Below the global fit, all models are run separately by sex and for six time periods: 1990, 1995, 2000, 2005, 2010, and 2016. During each fit all data on prevalence, incidence, remission (ie, cure rate) and mortality are forced to be internally consistent. For most diseases, the bulk of data on prevalence or incidence is at the disease level with fewer studies providing data on the proportions of cases of disease in each of the sequelae defined for the disease. The proportions in each sequela are pooled using DisMod-MR 2.1 or meta-analysis, or derived from analyses of patient-level datasets. The multiplication of prevalent cases for each disease sequela and the appropriate disability weight produces YLD estimates that do not yet take into account comorbidity. To correct for comorbidity, these data are used in a simulation to create hypothetical individuals in each age, sex, location, and year combination who experience no, one, or multiple sequelae simultaneously. We assume that disability weights are multiplicative rather than additive as this avoids assigning a combined disability weight value in any individual to exceed 1, ie, be worse than a "year lost due to death". This comorbidity adjustment leads to an average scaling down of disease-specific YLDs ranging from about 2% in young children up to 17% in oldest ages.

All our estimates of causes of death are categorical: each death is assigned to a single underlying cause. This has the attractive property that all estimates add to 100%. For risks, we use a different, "counterfactual" approach, ie, answering the question: "what would the burden have been if the population had been exposed to a theoretical minimum level of exposure to a risk". Thus, we need to define what level of exposure to a risk factor leads to the lowest amount of disease. We then analyse data

on the prevalence of exposure to a risk and derive relative risks for any risk-outcome pair for which we find sufficient evidence of a causal relationship. Prevalence of exposure is estimated in DisMod-MR 2.1, using spatiotemporal Gaussian Process Regression, or from satellite imagery in the case of ambient air pollution. Relative risk data are pooled using meta-analysis of cohort, case-control and/or intervention studies. For each risk and outcome pair, we evaluate the evidence and judge if the evidence falls into the categories of "convincing" or "probable" as defined by the World Cancer Research Fund.[5] From the prevalence and relative risk results, population attributable fractions are estimated relative to the theoretical minimum risk exposure level (TMREL). When we aggregate estimates for clusters of risks, eg, metabolic or behavioural risks, we use a multiplicative function rather than simple addition and take into account how much of each risk is mediated through another risk. For instance, some of the risk of high body mass index is directly onto stroke as an outcome but much of its impact is mediated through high blood pressure, high cholesterol, or high fasting plasma glucose, and we would not want to double count the mediated effects when we estimate aggregates across risk factors.[6]

Uncertainty is propagated throughout all these calculations by creating 1,000 values for each prevalence, death, YLL, YLD, or DALY estimate and performing aggregations across causes and locations at the level of each of the 1,000 values for all intermediate steps in the calculation. The lower and upper bounds of the 95% uncertainty interval are the 25th and 975th values of the ordered 1,000 values. For all age-standardised rates, GBD uses a standard population calculated as the non-weighted average across all countries of the percentage of the population in each five-year age group for the years 2010 to 2035 from the United Nations Population Division's World Population Prospects (2012 revision).[7,8]

GBD uses a composite indicator or sociodemographic development, SDI, which reflects the geometric mean of normalised values of a location's income per capita, the average years of schooling in the population 15 and over, and the total fertility rate. Countries and territories are grouped into five quintiles of high, high-middle, middle, low-middle, and low SDI based on their 2016 values.[2]

The fatal and non-fatal write-ups have been published before as part of the GBD papers on causes of death and non-fatal estimates published in the Lancet weekly (see references [2,4,6,7]).

1 GBD 2015 Neurological Disorders Collaborator Group. Global, regional, and national burden of neurological disorders during 1990-2015: a systematic analysis for the Global Burden of Disease Study 2015. *Lancet Neurol* 2017; **16**: 877–97.

2 GBD 2016 Mortality Collaborators. Global, regional, and national under-5 mortality, adult mortality, age-specific mortality, and life expectancy, 1970-2016: a systematic analysis for the Global Burden of Disease Study 2016. *Lancet Lond Engl* 2017; **390**: 1084–150.

3 Salomon JA, Haagsma JA, Davis A, *et al.* Disability weights for the Global Burden of Disease 2013 study. *Lancet Glob Health* 2015; **3**: e712-723.

4 GBD 2016 Causes of Death Collaborators. Global, regional, and national age-sex specific mortality for 264 causes of death, 1980-2016: a systematic analysis for the Global Burden of Disease Study 2016. *Lancet Lond Engl* 2017; **390**: 1151–210.

5 American Institute for Cancer Research. Food, nutrition, physical activity, and the prevention of cancer: a global perspective. Washington, DC: American Institute for Cancer Research, 2007.

6 GBD 2016 Risk Factors Collaborators. Global, regional, and national comparative risk assessment of 84 behavioural, environmental and occupational, and metabolic risks or clusters of risks, 1990-2016: a systematic analysis for the Global Burden of Disease Study 2016. *Lancet Lond Engl* 2017; **390**: 1345–422.

7 GBD 2013 Mortality and Causes of Death Collaborators. Global, regional, and national age-sex specific all-cause and cause-specific mortality for 240 causes of death, 1990-2013: a systematic analysis for the Global Burden of Disease Study 2013. *Lancet Lond Engl* 2015; **385**: 117–71.

8 United Nations Department of Economics and Social Affairs Population Division. World Population Prospects: The 2012 Revision. http://esa.un.org/unpd/wpp/Documentation/publications.htm (accessed Nov 4, 2014).

GATHER compliance table

GATHER checklist of information that should be included in reports of global health estimates, with description of compliance and location of information for GBD 2016 (Table 1).

Table 1. GATHER check List

#	GATHER checklist item	Description of compliance	Reference
**Objectives and funding**			
1	Define the indicators, populations, and time periods for which estimates were made.	Narrative provided in paper and appendix describing indicators, definitions, and populations	Main text (Methods) and appendix
2	List the funding sources for the work.	Funding sources listed in paper	Summary (Funding)
**Data Inputs**			
For all data inputs from multiple sources that are synthesised as part of the study:			
3	Describe how the data were identified and how the data were accessed.	Narrative description of data seeking methods provided	Main text (Methods) and appendix
4	Specify the inclusion and exclusion criteria. Identify all ad-hoc exclusions.	Narrative about inclusion and exclusion criteria by data type provided; ad hoc exclusions in cause-specific write-ups	Main text (Methods) and appendix
5	Provide information on all included data sources and their main characteristics. For each data source used, report reference information or contact name/institution, population represented, data collection method, year(s) of data collection, sex and age range, diagnostic criteria or measurement method, and sample size, as relevant.	An interactive, online data source tool that provides metadata for data sources by component, geography, cause, risk, or impairment has been developed	Online data citation tools: http://ghdx.healthdata.org/gbd-2016
6	Identify and describe any categories of input data that have potentially important biases (e.g., based on characteristics listed in item 5).	Summary of known biases by cause included in appendix	Appendix
For data inputs that contribute to the analysis but were not synthesised as part of the study:			
7	Describe and give sources for any other data inputs.	Included in online data source tool	http://ghdx.healthdata.org/gbd-2016
For all data inputs:			
8	Provide all data inputs in a file format from which data can be efficiently extracted (e.g., a spreadsheet as opposed to a PDF), including all relevant meta-data listed in item 5. For any	Downloads of input data available through online tools, including data	Online data visualisation tools, data query tools, and

	data inputs that cannot be shared due to ethical or legal reasons, such as third-party ownership, provide a contact name or the name of the institution that retains the right to the data.	visualisation tools and data query tools; input data not available in tools will be made available upon request	the Global Health Data Exchange
**Data analysis**			
9	Provide a conceptual overview of the data analysis method. A diagram may be helpful.	Flow diagrams of the overall methodological processes, as well as cause-specific modelling processes, have been provided	Main text (Methods) and appendix
10	Provide a detailed description of all steps of the analysis, including mathematical formulae. This description should cover, as relevant, data cleaning, data pre-processing, data adjustments and weighting of data sources, and mathematical or statistical model(s).	Flow diagrams and corresponding methodological write-ups for each cause, as well as the databases and modelling processes, have been provided	Main text (Methods) and appendix
11	Describe how candidate models were evaluated and how the final model(s) were selected.	Provided in the methodological write-ups	Appendix
12	Provide the results of an evaluation of model performance, if done, as well as the results of any relevant sensitivity analysis.	Provided in the methodological write-ups	Appendix
13	Describe methods for calculating uncertainty of the estimates. State which sources of uncertainty were, and were not, accounted for in the uncertainty analysis.	Appendix	Appendix
14	State how analytic or statistical source code used to generate estimates can be accessed.	Appendix	http://ghdx.healthdata.org/gbd-2016-code
**Results and Discussion**			
15	Provide published estimates in a file format from which data can be efficiently extracted.	GBD 2016 results are available through online data visualisation tools, the Global Health Data Exchange, and the online data query tool	Main text, and online data tools (data visualisation tools, data query tools, and the Global Health Data Exchange)
16	Report a quantitative measure of the uncertainty of the estimates (e.g. uncertainty intervals).	Uncertainty intervals are provided with all results	Main text, appendix, and online data tools (data visualisation tools, data query tools, and the Global Health Data Exchange)
17	Interpret results in light of existing evidence. If updating a previous set of estimates, describe the reasons for changes in estimates.	Discussion of methodological changes between GBD rounds provided in the narrative of the manuscript and appendix	Main text (Methods and Discussion) and appendix
18	Discuss limitations of the estimates. Include a discussion of any modelling assumptions or data limitations that affect interpretation of the estimates.	Discussion of limitations provided in the narrative of the main paper, as well as in the methodological write-ups	Main text (Limitations) and appendix

		in the appendix	

Epilepsy mortality

Flowchart

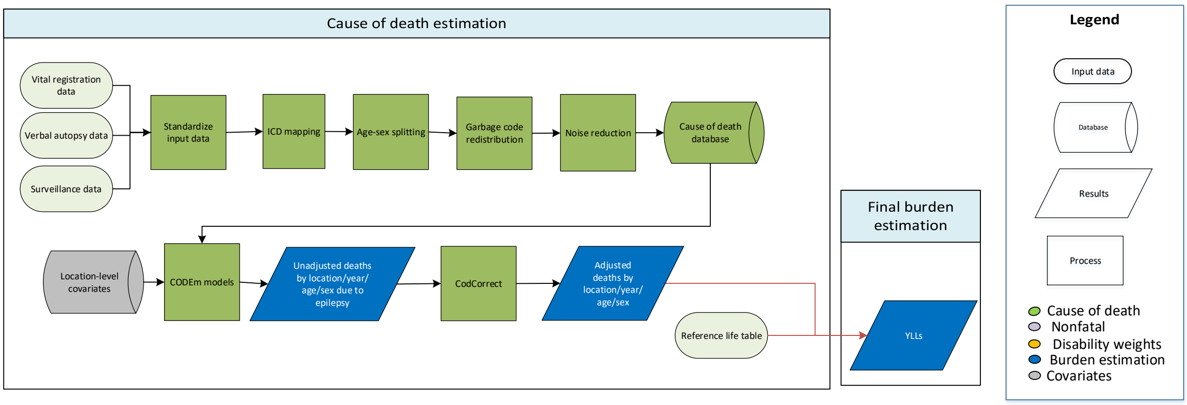

Input data

Data used to estimate epilepsy mortality included vital registration (VR), verbal autopsy, and China mortality surveillance data from the cause of death (COD) database. Our outlier criteria were to exclude data points that (1) were implausibly high or low relative to global or regional patterns, (2) substantially conflicted with established age or temporal patterns, or (3) significantly conflicted with other data sources based from the same locations or locations with similar characteristics (ie, Socio-demographic Index).

Based on these criteria, we excluded ICD-9 BTL data for Sri Lanka, Fiji, and Kiribati as the estimates varied from year to year between zero and high values. We also excluded the Survey of Causes of Death Data and Medical Certification of Cause of Death Data for India, as these data types were not consistent with the Sample Registration System Data and would have led to discontinuities in our estimates over time.

Modelling strategy

The standard CODEm modelling approach was applied to estimate deaths due to epilepsy. Separate models were conducted for male and female mortality, and the age range for both models was 28 days–95+ years. For GBD 2016, the health systems access covariate was replaced with the healthcare access and quality index covariate. There were no other substantial changes for GBD 2016. The covariates used are displayed below (Table 2).

Table 2. List of covariates

Level	Covariate	Direction
1	pig meat consumption (kcal per capita)	+
	pigs (per capita)	+
	SEV scalar: epilepsy	+

	mean systolic blood pressure (mmHg)	+
2	health access and quality index	-
	mean body mass index	+
	mean serum total cholesterol (mmol/L)	+
3	cumulative cigarettes (10 years)	+
	cumulative cigarettes (5 years)	+
	education (years per capita)	-
	log LDI (per capita)	-
	Socio-demographic Index	-

Epilepsy Impairment

Flowchart

Epilepsy

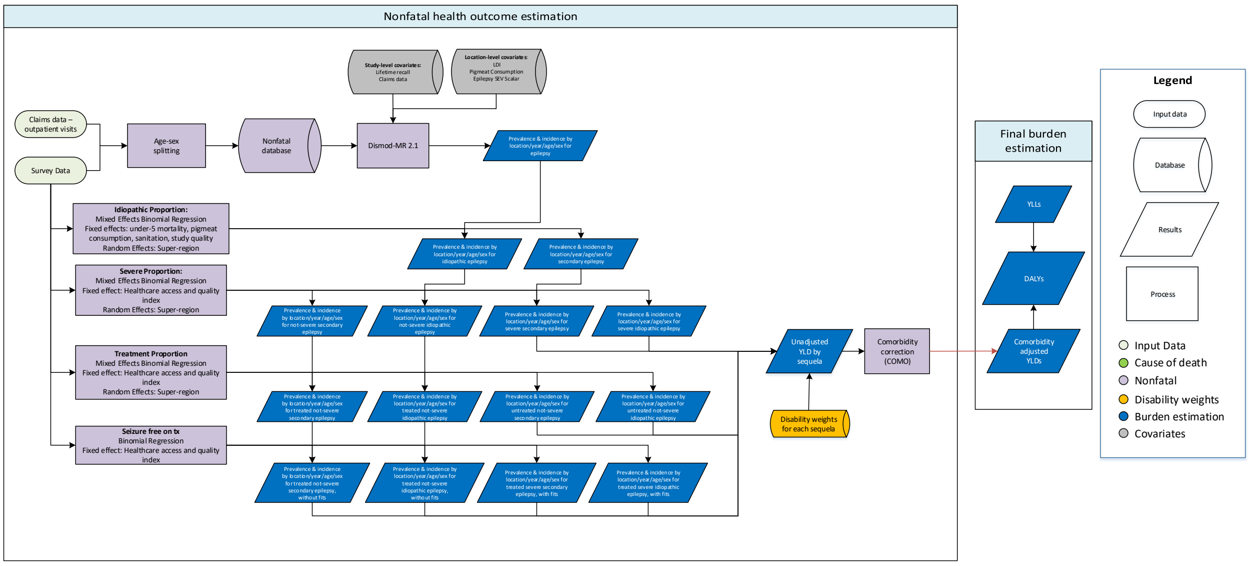

Case definition

Since GBD 2013, we have used the following definitions from the "Guidelines for Epidemiologic Studies on Epilepsy": 1) Epilepsy: a condition characterized by recurrent (two or more) epileptic seizures, unprovoked by any immediate identified cause, and 2) "Active" epilepsy: a prevalent case of active epilepsy is defined as a person with epilepsy who has had at least one epileptic seizure in the previous five years, regardless of antiepileptic drug (AED) treatment. We also use the following ICD-10 codes for epilepsy: G40 (Neuro, epilepsy, total) and G41 (Neuro, epilepsy, status epilepticus). We defined severe epilepsy as having seizures one or more times per month.

Input data

Model inputs

For GBD 2016, we conducted a systematic review covering 10/1/2014 to 10/17/2016 using the following search string:

("2014/10/01"[PDAT] : "2016"[PDAT]) AND ("epilepsy"[MeSH Terms] OR "epilepsy, partial, motor"[MeSH Terms] OR "epilepsy, benign neonatal"[MeSH Terms] OR "epilepsy, reflex"[MeSH Terms] OR "myoclonic epilepsy, juvenile"[MeSH Terms] OR "epilepsy, frontal lobe"[MeSH Terms] OR "epilepsy, complex

partial"[MeSH Terms] OR "epilepsy, post-traumatic"[MeSH Terms] OR "epilepsy, temporal lobe"[MeSH Terms] OR "epilepsy, absence"[MeSH Terms] OR "epilepsy, tonic-clonic"[MeSH Terms] OR "epilepsies, myoclonic"[MeSH Terms] OR "epilepsies, partial"[MeSH Terms] OR epilepsy[Title/Abstract]) AND (incidence[Title/Abstract] OR prevalence[Title/Abstract]) NOT(animals[MeSH] NOT humans[MeSH]).

Of 952 hits, 32 were marked for extraction. Additional data-seeking efforts also led to the addition of 12 more sources on epilepsy prevalence in India subnationals. A flow chart documenting the review is displayed below.

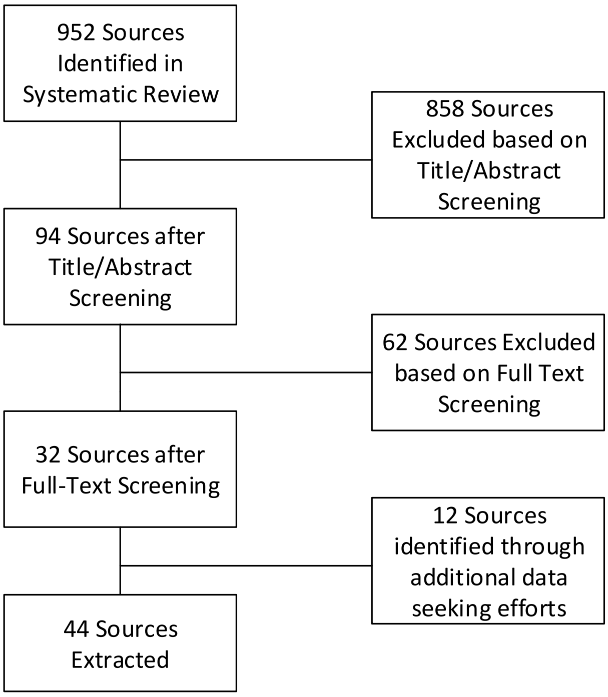

We included representative, population-based surveys that reported of prevalence, incidence, remission rate, excess mortality rate, relative risk of mortality, standardised mortality ratio, or with-condition mortality rate. We excluded studies with no clearly defined sample (eg, among clinic attenders or patient organisation members with non-specific or non-representative catchment area). The table below (Table 3) details the model inputs used to estimate the epilepsy impairment.

Table 3. Model inputs used to estimate epilepsy impairment

	Prevalence	Incidence	Mortality risk
Studies	317	81	23
Countries/subnationals	192	51	23
GBD world regions	20	15	10

The inputs for the regressions used to split the epilepsy impairment envelope were also updated for GBD 2016. These regressions are used to determine the proportion of epilepsy that is primary or idiopathic, the proportion of epilepsy that is severe (one or more seizures per month), the proportion of epilepsy that is untreated (the treatment gap), and the proportion of treated epilepsy that is treated without seizures (no seizures reported in the preceding year).

For GBD 2016, a new systematic review was conducted covering 1/1/2006 to 10/17/2016 using the search term:

("2006"[PDAT] : "2016"[PDAT]) AND ("epilepsy"[MeSH Terms] OR "epilepsy, partial, motor"[MeSH Terms] OR "epilepsy, benign neonatal"[MeSH Terms] OR "epilepsy, reflex"[MeSH Terms] OR "myoclonic epilepsy, juvenile"[MeSH Terms] OR "epilepsy, frontal lobe"[MeSH Terms] OR "epilepsy, complex partial"[MeSH Terms] OR "epilepsy, post-traumatic"[MeSH Terms] OR "epilepsy, temporal lobe"[MeSH Terms] OR "epilepsy, absence"[MeSH Terms] OR "epilepsy, tonic-clonic"[MeSH Terms] OR "epilepsies, myoclonic"[MeSH Terms] OR "epilepsies, partial"[MeSH Terms] OR epilepsy[Title/Abstract]) AND (incidence[Title/Abstract] OR prevalence[Title/Abstract] OR epidemiology[Title/Abstract]) AND (sever*[Title/Abstract] OR treated[Title/Abstract] OR "drug resistant epilepsy"[MeSH] OR "treatment resistant"[Title/Abstract] OR proportion[Title/Abstract] OR "clinical characteristics"[Title/Abstracts] OR "treatment gap"[Title/Abstract]) NOT(animals[MeSH] NOT humans[MeSH])

Of 1,234 hits, 37 were marked for extraction. Additional data-seeking efforts also led to the addition of five more sources on the treatment gap in India subnationals. A flow chart documenting the review is displayed below.

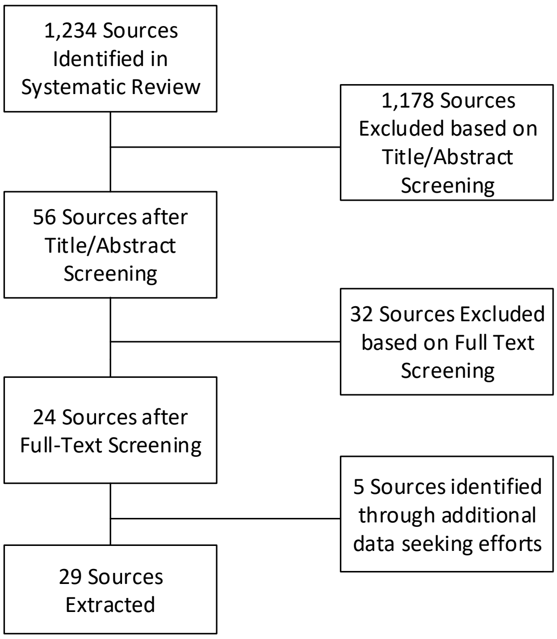

Severity splits & disability weights

To adjust for differences in methodological quality, all prevalence studies included in GBD were scored according to a modified version (dichotomised variables) of published methodological quality criteria for epilepsy epidemiological studies, taking into account the representativeness of the population of interest (representative of country or community versus selected population), quality of sampling (random sample of the population of interest versus not random sample), recall period (one-year prevalence versus other recall period), participation rate (≥ 70% versus <70%), survey method (face to face with epilepsy expert or trained interviewer versus other), validation of diagnostic instrument (sensitivity or specificity ≥ 70% versus < 70% or no validation). In DisMod-MR these methodological variables were evaluated for a systematic difference and corrected accordingly.

Table 4 illustrates the severity levels, descriptions, and disability weights associated with epilepsy. These are calculated using regressions from literature (ie, frequency of seizures).

Table 4. Severity levels, descriptions, and disability weights associated with epilepsy

Severity level	Lay description	Disability weights (95% CI)
severe (seizures >= once per month)	This person has sudden seizures one or more times each month, with violent muscle contractions and stiffness, loss of consciousness, and loss of urine or bowel control. Between seizures the person has memory loss and difficulty concentrating.	0.552 (0.375–0.71)
less severe (seizures < once per month)	This person has sudden seizures two to five times a year, with violent muscle contractions and stiffness, loss of consciousness, and loss of urine or bowel control.	0.263 (0.173–0.367)
Treated without fits	This person has a chronic disease that requires medication every day and causes some worry but minimal interference with daily activities.	0.049 (0.031–0.072)

Modelling strategy

We modelled the prevalence of epilepsy in two steps: first, we created an epilepsy impairment envelope. Second, we split the envelope into primary (or idiopathic) and secondary epilepsies. Each of these were subdivided into "severe" (on average one or more fits per month) and "non-severe." Non-severe cases were subdivided into "treated" and "un-treated." Finally, "treated" cases were divided into "treated cases with fits" (between one and 11 fits on average in the preceding year) and "treated cases without fits" (no fits reported in the preceding year).

In the first step, we used the DisMod-MR tool for the epilepsy impairment envelope to model a consistent fit between incidence, prevalence, remission, and standardised mortality ratio data while using meta-regression to correct data points with non-reference study quality characteristics. We found no systematic bias for the covariate "non-standard case definition" indicating studies that did not define "active epilepsy" and additionally the covariate was not significant as a "z-cov", which acts as a multiplier applied to the standard error and thus results in these data points being given less weight in the analysis than the "reference" data points. Therefore, we excluded this covariate from the model. We also included data on lifetime prevalence and therefore added a covariate on lifetime prevalence data points. We also included country-level covariates on prevalence for the SEV epilepsy scalar, which summarises the epilepsy risk exposure level for each country, and pig meat consumption per capita, which is used as a proxy for the level of neurocysticercosis, a common cause of secondary epilepsy. We included cause-specific mortality rate (CSMR) results from the epilepsy mortality model as input data to the DisMod model. Where age-specific prevalence data were available, we calculated excess mortality rate (EMR) from prevalence and CSMR. We included the log of the lag distributed income (LDI) as a covariate on EMR to account for lower mortality in developed countries. We included Bayesian priors on remission to account for the scarcity of remission data. We set bounds on remission from 0 to 0.25 from age 0 to 60 and 0 to 0.05 from age 61 to 100. Table 5 indicates the covariates used in the estimation process, as well as parameters, betas, and exponentiated betas.

Table 5. Covariates used in the estimation process

Measure	Variable Name	Beta	Exponentiated
prevalence	Recall Lifetime	0.18 (0.15 – 0.22)	1.20 (1.17–1.24)
prevalence	All MarketScan, year 2000	-0.89 (-0.94 – -0.83)	0.41 (0.39–0.43)
prevalence	All MarketScan, year 2010	-0.43 (-0.47 – -0.37)	0.65 (0.62–0.69)
prevalence	All MarketScan, year 2012	-0.35 (-0.41 – -0.30)	0.70 (0.67–0.74)
prevalence	Pig Meat Consumption (kg per capita)	0.0054 (0.00012 – 0.015)	1.01 (1.00–1.02)
prevalence	Log-transformed age-standardized SEV scalar for epilepsy	0.79 (0.75-0.90)	2.21 (2.12-2.46)
excess mortality rate	LDI (I$ per capita)	-0.55 (-1 – -0.1)	0.58 (1.37–0.90)

In the second step, we used a mixed-effects generalized linear model (binomial family) to predict the proportion of idiopathic epilepsy, the proportion of severe epilepsy, the proportion of treated epilepsy, and the proportion of epilepsy that is treated without fits.

Because not all of the data on the proportion of idiopathic epilepsy use optimal case-finding methods (using CT scans or MRIs in addition to EEGs in order to diagnose secondary epilepsy), for GBD 2016 we decided to add a covariate to crosswalk studies with non-optimal case-finding methods to those with adequate methods. The regression for the proportion of epilepsy that is idiopathic therefore has fixed effects on this study quality covariate as well as the under-5 mortality rate, the log of pig meat consumption (per capita), and the proportion of a country with access to proper sanitation, as well as a random effect on super-region.

We used similar models to predict the proportion of severe epilepsy and treatment gap based on the reported proportions extracted from the systematic review. To predict the proportion of severe epilepsy and the treatment gap, we used mixed-effects models with a fixed effect on the log of HAQ Index and a random effect on super-region.

For GBD 2015, a meta-analysis was used to generate two different pooled estimates for proportion of treated epilepsy that is seizure-free in developing and developed countries, as there were not enough data to run a regression. However, for GBD 2016 the expanded dataset allowed for the implementation of a generalised linear model (binomial family) to generate predictions for the proportion of treated epilepsy that is seizure-free. We used a fixed effect on the log of HAQ Index.

We tested a fixed effect on Socio-demographic Index (SDI) and random effects on region and country in different models, but they did not improve the model. We generated 1,000 draws of country-specific estimates for each year between 1980 and 2016 for each of the models. Table 6 shows the betas from these regressions.

Table 6. Estimated parameters (beta) with standard errors from regression models

Regression	covariate	beta	SE
Idiopathic	Under-5 mortality	-65.82	7.50
Idiopathic	Pig meat consumption	-0.12	0.02
Idiopathic	Sanitation	0.45	0.16

Idiopathic	Study quality	0.88	0.07
Severe	HAQ Index	-2.15	0.24
Treatment gap	HAQ Index	-3.17	0.18
Treated without fits	HAQ Index	3.65	0.21

Definition of GBD super-regions and regions

Table 7. GBD Super-regions and Regions

Super-region	Region_name
East Asia, Southeast Asia and Oceania	East Asia
	Southeast Asia
	Oceania
Central Asia, Central Europe, Eastern Europe	Central Asia
	Central Europe
	Eastern Europe
High-income	High-income Asia Pacific
	Australasia
	Western Europe
	High-income North America
	Southern Latin America
Latin America & Caribbean	Caribbean
	Andean Latin America
	Central Latin America
	Tropical Latin America
North Africa and Middle East	North Africa and Middle East
South Asia	South Asia
Sub-Saharan Africa	Central sub-Saharan Africa
	Eastern sub-Saharan Africa
	Southern sub-Saharan Africa
	Western sub-Saharan Africa

Count of data sources used in nonfatal modelling for epilepsy by 21 regions in 2016

Table 8. Count of data sources used in nonfatal modelling for epilepsy by 21 regions in 2016

Region_name	Incidence	Prevalence	Remission	Mortality	Hospital_claims
East Asia	3	40	0	4	0
Southeast Asia	2	5	0	0	0
Oceania	0	0	0	0	0
Central Asia	0	2	0	0	0
Central Europe	2	4	0	0	0
Eastern Europe	2	4	0	1	0
High-income Asia Pacific	2	6	0	0	0
Australasia	0	2	0	1	0
Western Europe	42	75	0	10	0
Southern Latin America	1	6	1	1	0
High-income North America	7	16	1	4	3
Caribbean	0	2	0	0	0
Andean Latin America	3	9	1	1	0
Central Latin America	0	12	0	0	0
Tropical Latin America	1	8	0	0	0
North Africa and Middle East	5	23	0	0	0
South Asia	4	36	0	2	0
Central sub-Saharan Africa	0	2	0	0	0
Eastern sub-Saharan Africa	4	25	0	1	0
Southern sub-Saharan Africa	1	5	0	0	0
Western sub-Saharan Africa	3	37	0	3	0
**Total**	**82**	**319**	**3**	**28**	**3**

Sources Used for Epilepsy Regressions

Idiopathic Regression

Huang DH, Zheng JO, Chen J, Yu L. Treatment gaps of epilepsy and retention rates of sodium valproate in rural Guangxi, China. <i>Genet Mol Res</i>. 2014; 13(3): 6202-12.
Kong ST, Ho CS, Ho PC, Lim SH. Prevalence of drug resistant epilepsy in adults with epilepsy attending a neurology clinic of a tertiary referral hospital in Singapore. <i>Epilepsy Res</i>. 2014; 108(7): 1253-62.
Choudhary A, Gulati S, Sagar R, Kabra M, Sapra S. Behavioral comorbidity in children and adolescents with epilepsy. <i>J Clin Neurosci</i>. 2014; 21(8): 1337-40.
Stefan H, May TW, Pfafflin M, Brandt C, Furatsch N, Schmitz B, Wandschneider B, Kretz R, Runge U, Geithner J, Karakizlis C, Rosenow F, Kerling F. Epilepsy in the elderly: comparing clinical characteristics with younger patients. <i>Acta Neurol Scand</i>. 2014; 129(5): 283-93.
Kim DW, Lee SY, Chung SE, Cheong HK, Jung KY. Clinical characteristics of patients with treated epilepsy in Korea: a nationwide epidemiologic study. <i>Epilepsia</i>. 2014; 55(1): 67-75.
Pandey S, Singhi P, Bharti B. Prevalence and treatment gap in childhood epilepsy in a north Indian city: a community-based study. <i>J Trop Pediatr</i>. 2014; 60(2): 118-23.
Joseph N, Kumar GS, Nelliyanil M. Pattern of seizure cases in tertiary care hospitals in Karnataka state of India. <i>Ann Indian Acad Neurol</i>. 2013; 16(3): 347-51.
Torres-Ferrus M, Toledo M, Gonzalez-Cuevas M, Sero-Ballesteros L, Santamarina E, Raspall-Chaure M, Sueiras-Gil M, Cambrodi-Masip R, Sarria S, Alvarez-Sabin J, Salas-Puig J. [Aetiology and treatment of epilepsy in a series of 1,557 patients]. <i>Rev Neurol</i>. 2013; 57(7): 306-12.
Tanaka A, Akamatsu N, Shouzaki T, Toyota T, Yamano M, Nakagawa M, Tsuji S. Clinical characteristics and treatment responses in new-onset epilepsy in the elderly. <i>Seizure</i>. 2013; 22(9): 772-5.
Chong J, Hesdorffer DC, Thurman DJ, Lopez D, Harris RB, Hauser WA, Labiner ET, Velarde A, Labiner DM. The prevalence of epilepsy along the Arizona-Mexico border. <i>Epilepsy Res</i>. 2013; 105(2-Jan): 206-15.
Nickels KC, Grossardt BR, Wirrell EC. Epilepsy-related mortality is low in children: a 30-year population-based study in Olmsted County, MN. <i>Epilepsia</i>. 2012; 53(12): 2164-71.
Hunter E, Rogathi J, Chigudu S, Jusabani A, Jackson M, McNally R, Gray W, Whittaker RG, Iqbal A, Birchall D, Aris E, Walker R. Prevalence of active epilepsy in rural Tanzania: a large community-based survey in an adult population. <i>Seizure</i>. 2012; 21(9): 691-8.
Garcia-Martin G, Perez-Errazquin F, Chamorro-Munoz MI, Romero-Acebal M, Martin-Reyes G, Dawid-Milner MS. Prevalence and clinical characteristics of epilepsy in the South of Spain. <i>Epilepsy Res</i>. 2012; 102(1-2): 100-8.
Zhao Y-H, Zhang Q, Long N, Yang C, Hong J, Mu L, Zhou D. Prevalence of epilepsy and alcohol-related risk in Zayul County, Tibet Autonomous Region in China: an initial survey. <i>Epilepsy Behav</i>. 2010; 19(4): 635-8.
Suastegui R, Gutierrez J, Ramos R, Bouchan S, Navarrete H, Ruiz J, Plascencia N, Jauri S, Leon C, Castillo V, Ojeda EA. [Clinical characteristics of the late-onset epilepsy in Mexico to the beginning of the new millennium: 455 cases]. <i>Rev Invest Clin</i>. 2009; 61(5): 354-63.
Tse E, Hamiwka L, Sherman EM, Wirrell E. Social skills problems in children with epilepsy: prevalence, nature and predictors. <i>Epilepsy Behav</i>. 2007; 11(4): 499-505.
Dura Trave T, Yoldi Petri ME. [Epilepsy and epileptic syndromes among primary school children (6-12 years)]. <i>An Pediatr (Barc)</i>. 2007; 66(1): 11-6.
Oun A, Haldre S, Magi M. Use of antiepileptic drugs in Estonia: an epidemiologic study of adult epilepsy. <i>Eur J Neurol</i>. 2006; 13(5): 465-70.
Arruda WO. Etiology of epilepsy. A prospective study of 210 cases. <i>Arq Neuropsiquiatr</i>. 1991; 49(3): 251?4.
Valen‡a MM, Valen‡a LP. [Etiology of the epileptic seizures in Recife city, Brazil: study of 249 patients]. <i>Arq Neuropsiquiatr</i>. 2000; 58(4): 1064?72.

Kwong KL, Chak WK, Wong SN, So KT. Epidemiology of childhood epilepsy in a cohort of 309 Chinese children. <i>Pediatr Neurol</i>. 2001; 24(4): 276-82.

Ng KK, Ng PW, Tsang KL, Hong Kong Epilepsy Study Group. Clinical characteristics of adult epilepsy patients in the 1997 Hong Kong epilepsy registry. <i>Chin Med J (Engl)</i>. 2001; 114(1): 84-7.

Hui AC, Wong A, Wong HC, Man BL, Au-Yeung KM, Wong KS. Refractory epilepsy in a Chinese population. <i>Clin Neurol Neurosurg</i>. 2007; 109(8): 672-5.

Fong GCY, Mak W, Cheng TS, Chan KH, Fong JKY, Ho SL. A prevalence study of epilepsy in Hong Kong. <i>Hong Kong Med J</i>. 2003; 9(4): 252-7.

Pi X, Cui L, Liu A, Zhang J, Ma Y, Liu B, Cai C, Zhu C, Zhou T, Chen J, Zhou Z, Wang C, Li L, Li S, Wu J, Xiao B. Investigation of prevalence, clinical characteristics and management of epilepsy in Yueyang city of China by a door-to-door survey. <i>Epilepsy Res</i>. 2012; 101(1-2): 129-34.

Dechef G. Notions sur l',pid,miologie de l',pilepsie au Congo (Kinshasa). <i>Afr J Med Sci</i>. 1970; 1(3): 309-14.

Chaves-Sell F, Dubuisson-Schonemberg V. [Profile of epilepsy in a neurology clinic in Costa Rica]. <i>Rev Neurol</i>. 2001; 33(5): 411-13.

Pascual L¢pez MA, Pascual Gispert J, Rodr¡guez Rivera L, Rojas Ochoa F, Tejeiros A. [Epilepsy: epidemiological study in a child population]. <i>Bol Med Hosp Infant Mex</i>. 1980; 37(4): 811-21.

Arteaga-Rodr¡guez C, Ram¡rez-Ch vez J, Rodr¡guez-Rivera L, Morera-M,ndez F, Hern ndez-Fustes OJ. Aetiological factors of the epilepsies. <i>Rev Neurol</i>. 1998; 27: 427-30.

Nieto Barrera M. [Neuroepidemiology of epilepsies]. <i>An Esp Pediatr</i>. 1988; 29(Supp 33): 59-63.

Dur -Trav, T, Yoldi-Petri ME, Gallinas-Victoriano F. Incidence of epilepsies and epileptic syndromes among children in Navarre, Spain: 2002 through 2005. <i>J Child Neurol</i>. 2008; 23(8): 878-82.

Rakitin A, Liik M, Oun A, Haldre S. Mortality risk in adults with newly diagnosed and chronic epilepsy: a population-based study. <i>Eur J Neurol</i>. 2011; 18(3): 465-70.

Beilmann A, Napa A, H„marik M, S””t A, Talvik I, Talvik T. Incidence of childhood epilepsy in Estonia. <i>Brain Dev</i>. 1999; 21(3): 166-74.

Tekle-Haimanot R, Forsgren L, Ekstedt J. Incidence of epilepsy in rural central Ethiopia. <i>Epilepsia</i>. 1997; 38(5): 541-6.

Rantala H, Ingalsuo H. Occurrence and outcome of epilepsy in children younger than 2 years. <i>J Pediatr</i>. 1999; 135(6): 761-4.

Lomidze G, Kasradze S, Kvernadze D, Okujava N, Toidze O, de Boer HM, Dua T, Sander JW. The prevalence and treatment gap of epilepsy in Tbilisi, Georgia. <i>Epilepsy Res</i>. 2012; 98(2-3): 123-9.

Medina MT, Dur¢n RM, Mart¡nez L, Osorio JR, Estrada AL, Z£niga C, Cartagena D, Collins JS, Holden KR. Prevalence, incidence, and etiology of epilepsies in rural Honduras: the Salama Study. <i>Epilepsia</i>. 2005; 46(1): 124-31.

Goel D, Dhanai JS, Agarwal A, Mehlotra V, Saxena V. Neurocysticercosis and its impact on crude prevalence rate of epilepsy in an Indian community. <i>Neurol India</i>. 2011; 59(1): 37-40.

Olafsson E, Hauser WA. Prevalence of epilepsy in rural Iceland: a population-based study. <i>Epilepsia</i>. 1999; 40(11): 1529-34.

Olafsson E, Ludvigsson P, Gudmundsson G, Hesdorffer D, Kjartansson O, Hauser WA. Incidence of unprovoked seizures and epilepsy in Iceland and assessment of the epilepsy syndrome classification: a prospective study. <i>Lancet Neurol</i>. 2005; 4(10): 627-34.

Battaglia D, Rand¢ T, Deodato F, Bruccini G, Baglio G, Frisone MF, Pant¢ T, Tortorella G, Guzzetta F. Epileptic disorders with onset in the first year of life: neurological and cognitive outcome. <i>Eur J Paediatr Neurol</i>. 1999; 3(3): 95-103.

Gallitto G, Serra S, La Spina P, Postorino P, Lagan… A, Tripodi F, Gangemi S, Calabr• S, Savica R, Di Perri R, Beghi E, Musolino R. Prevalence and characteristics of epilepsy in the Aeolian islands. <i>Epilepsia</i>. 2005; 46(11): 1828-35.

Mwinzi SMG, Ruberti FR, Stewart JD. Epilepsy in the Kenyan Africa. <i>Med Afr Noire</i>. 1976; 23: 331-334.

Feksi AT, Kaamugisha J, Sander JW, Gatiti S, Shorvon SD. Comprehensive primary health care

antiepileptic drug treatment programme in rural and semi-urban Kenya. ICBERG (International Community-based Epilepsy Research Group). <i>Lancet</i>. 1991; 337(8738): 406-9.

Sridharan R, Radhakrishnan K, Ashok PP, Mousa ME. Epidemiological and clinical study of epilepsy in Benghazi, Libya. <i>Epilepsia</i>. 1986; 27(1): 60-5.

Andriantseheno LM, Andrianasy TF. The features of epilepsy in the Malagasy: a hospital study on 213 cases from the North Western part of Madagascar. <i>Afr J Neurol Sci</i>. 1997; 16( ): 28-33.

Medina MT, Rosas E, Rubio-Donnadieu F, Sotelo J. Neurocysticercosis as the main cause of late-onset epilepsy in Mexico. <i>Arch Intern Med</i>. 1990; 150(2): 325-7.

Uuriintuya M, Ulziibayar D, Bayarmaa D. Epilepsy in Mongolia. <i>Neurology Asia</i>. 2007; 12: 61-63.

Dada TO. Epilepsy in Lagos, Nigeria. <i>Afr J Med Sci</i>. 1970; 1( ): 161-184.

Danesi MA, Oni K. Features of partial epilepsy in Nigerians: a 3 year clinical and electroencephalographic study of 282 cases seen at the Lagos University Teaching Hospital. <i>Afr J Neurol Sci</i>. 1983; 2( ): 1-6.

Danesi MA. Acquired aetiological factors in Nigerian epileptics (an investigation of 378 patients). <i>Trop Geogr Med</i>. 1983; 35(3): 293-297.

Ojuawo A, Joiner KT. Childhood epilepsy in Ilorin, Nigeria. <i>East Afr Med J</i>. 1997; 74(2): 72-75.

Breteler M, de la Court A, Meinardi H, Hauser WA, Grobbee D, Hofman A. Prevalence of epilepsy in the elderly: the Rotterdam Study. <i>Epilepsia</i>. 1996; 37(2): 141-7.

Gaffo AL, Guill,n-Pinto D, Campos-Olaz bal P, Burneo JG. [Cysticercosis as the main cause of partial seizures in children in Peru]. <i>Rev Neurol</i>. 2004; 39(10): 924?6.

Collomb H, Girard PL, Konate S. L'e?pilepsie en milieu hospitalier a? Dakar. <i>Med Afr Noire</i>. 1976; 23: 299-304.

Lee WL, Low PS, Murugasu B, Rajan U. Epidemiology of epilespy in Singapore children. <i>Neurol J Southeast Asia</i>. 1997; 2: 31-35.

Larsson K, Eeg-Olofsson O. A population based study of epilepsy in children from a Swedish county. <i>Eur J Paediatr Neurol</i>. 2006; 10(3): 107-13.

Adel"w C, Andell E, Amark P, Andersson T, Hellebro E, Ahlbom A, Tomson T. Newly diagnosed single unprovoked seizures and epilepsy in Stockholm, Sweden: First report from the Stockholm Incidence Registry of Epilepsy (SIRE). <i>Epilepsia</i>. 2009; 50(5): 1094-101.

Velioglu SK, Bakirdemir M, Can G, Topbas M. Prevalence of epilepsy in northeast Turkey. <i>Epileptic Disord</i>. 2010; 12(1): 22-37.

Matuja WB. Aetological factors in Tanzanian epileptics. <i>East Afr Med J</i>. 1989; 66(5): 343-348.

Dent W, Helbok R, Matuja WBP, Scheunemann S, Schmutzhard E. Prevalence of active epilepsy in a rural area in South Tanzania: a door-to-door survey. <i>Epilepsia</i>. 2005; 46(12): 1963-9.

Tuan NA, Cuong LQ, Allebeck P, Chuc NTK, Persson HE, Tomson T. The prevalence of epilepsy in a rural district of Vietnam: a population-based study from the EPIBAVI project. <i>Epilepsia</i>. 2008; 49(9): 1634-7.

Chuke PO, Muras J. Experience in epilepsy in Lusaka. <i>Med J Zambia</i>. 1977; 11: 65-70.

Levy LF. Epilepsy in Rhodesia, Zambia, and Malawi. <i>Afr J Med Sci</i>. 1970; 1( ): 291-203.

Khedr EM, Shawky OA, Ahmed MA, Elfetoh NA, Al Attar G, Ali AM, Kandil MR, Farweez H. A community based epidemiological study of epilepsy in Assiut Governorate/Egypt. <i>Epilepsy Res</i>. 2013; 103(3-Feb): 294-302.

Al Rajeh S, Awada A, Bademosi O, Ogunniyi A. The prevalence of epilepsy and other seizure disorders in an Arab population: a community-based study. <i>Seizure</i>. 2001; 10(6): 410-4.

Ablah E, Hesdorffer DC, Liu Y, Paschal AM, Hawley S, Thurman D, Hauser WA. Prevalence of epilepsy in rural Kansas. <i>Epilepsy Res</i>. 2014; 108(4): 792-801.

El-Tallawy HN, Farghaly WM, Shehata GA, Abdel-Hakeem NM, Rageh TA, Abo-Elftoh NA, Hegazy A, Badry R. Epidemiology of epilepsy in New Valley Governorate, Al Kharga District, Egypt. <i>Epilepsy Res</i>. 2013; 104(2-Jan): 167-74.

<p>Saha, S. P., Bhattachrya, S., Roy, B. K., Basu, A., Roy, T., Maity, B., & Das, S. K. (2008). A

prospective incidence study of epilepsy in a rural community of West-Bengal, India. Neurology Asia, 13, 41-48.</p>

Josipovic-Jelic Z, Sonicki Z, Soljan I, Demarin V, Collaborative Group for Study of Epilepsy Epidemiology in Sibenik-Knin County, Croatia. Prevalence and socioeconomic aspects of epilepsy in the Croatian county of Sibenik-Knin: community-based survey. <i>Epilepsy Behav</i>. 2011; 20(4): 686-90.

Gaily E, Lommi M, Lapatto R, Lehesjoki AE. Incidence and outcome of epilepsy syndromes with onset in the first year of life: A retrospective population-based study. <i>Epilepsia</i>. 2016; nan.

Nilsson G, Fernell E, Arvidsson T, Neville B, Olsson I, Gillberg C. Prevalence of Febrile Seizures, Epilepsy, and Other Paroxysmal Attacks in a Swedish Cohort of 4-Year-Old Children. <i>Neuropediatrics</i>. 2016; nan.

El-Tallawy HN, Farghaly WM, Rageh TA, Shehata GA, Metwally NA, Badry R, Sayed MA, Abdelwarith AM, Kandil MR, Hamed MA, Mohamed KO, Tohamy AM. Spectrum of epilepsy - prevalence, impact, and treatment gap: an epidemiological study from Al-Quseir, Egypt. <i>Neuropsychiatr Dis Treat</i>. 2016; 12: 1111-8.

Serrano-Castro PJ, Mauri-Llerda JA, Hernandez-Ramos FJ, Sanchez-Alvarez JC, Parejo-Carbonell B, Quiroga-Subirana P, Vazquez-Gutierrez F, Santos-Lasaosa S, Mendez-Lucena C, Redondo-Verge L, Tejero-Juste C, Morandeira-Rivas C, Sancho-Rieger J, Matias-Guiu J. Adult Prevalence of Epilepsy in Spain: EPIBERIA, a Population-Based Study. <i>ScientificWorldJournal</i>. 2015; 2015: 602710.

Hara HS, Gupta A, Singh M, Raj R, Singh H, Pawar G, Hara PK, Singh J. Epilepsy in Punjab (India): A Population-Based Epidemiologic Study. <i>Neuroepidemiology</i>. 2015; 45(4): 273-81.

Hashem S, Al-Kattan M, Ibrahim SY, Shalaby NM, Shamloul RM, Farrag M. Epilepsy prevalence in Al-Manial Island, Egypt. A door-to-door survey. <i>Epilepsy Res</i>. 2015; 117: 133-7.

Fawi G, Khedr EM, El-Fetoh NA, Thabit MN, Abbass MA, Zaki AF. Community-based epidemiological study of epilepsy in the Qena governorate in Upper Egypt, a door-to-door survey. <i>Epilepsy Res</i>. 2015; 113: 68-75.

San-Juan D, Alvarado-Leon S, Barraza-Diaz J, Davila-Avila NM, Ruiz AH, Anschel DJ. Prevalence of epilepsy, beliefs and attitudes in a rural community in Mexico: A door-to-door survey. <i>Epilepsy Behav</i>. 2015; 46: 140-3.

Syvertsen M, Nakken KO, Edland A, Hansen G, Hellum MK, Koht J. Prevalence and etiology of epilepsy in a Norwegian county-A population based study. <i>Epilepsia</i>. 2015; 56(5): 699-706.

Bourrous M, Elibrahimi I, Draiss G, Safini F, Amine M, Bouskraoui M. [Characteristics of the children with epilepsy followed in the Marrakech University Hospital]. <i>Rev Neurol (Paris)</i>. 2010; 166(11): 921-6.

Wright J, Pickard N, Whitfield A, Hakin N. A population-based study of the prevalence, clinical characteristics and effect of ethnicity in epilepsy. <i>Seizure</i>. 2000; 9(5): 309-13.

Muir TM, Bradley A, Wood SF, Murray GD, Brodie MJ. An audit of treated epilepsy in Glasgow. West of Scotland Epilepsy Research Group. <i>Seizure</i>. 1996; 5(1): 41-6.

Aziz H, G•vener A, Akhtar SW, Hasan KZ. Comparative epidemiology of epilepsy in Pakistan and Turkey: population-based studies using identical protocols. <i>Epilepsia</i>. 1997; 38(6): 716-22.

Tran D-S, Odermatt P, Le T-O, Huc P, Druet-Cabanac M, Barennes H, Strobel M, Preux P-M. Prevalence of epilepsy in a rural district of central Lao PDR. <i>Neuroepidemiology</i>. 2006; 26(4): 199-206.

Okuma T, Kumashiro H. Natural history and prognosis of epilepsy: report of a multi-institutional study in Japan. The group for the study of prognosis of epilepsy in Japan. <i>Epilepsia</i>. 1981; 22(1): 35-53.

Manonmani V, Tan CT. A study of newly diagnosed epilepsy in Malaysia. <i>Singapore Med J</i>. 1999; 40(1): 32-5.

Andriantseheno LM, Ralaizandriny D. Pr,valence communautaire de l',pilepsie chez les Malgaches. <i>Epilepsies</i>. 2004; 16(2): 83-6.

Farnarier G, Diop S, Coulibaly B, Arborio S, Dabo A, Diakite M, Traore S, Banou A, Nimaga K, Vaz T, Doumbo O. [Onchocerciasis and epilepsy. Epidemiological survey in Mali]. <i>Med Trop (Mars)</i>. 2000; 60(2): 151-5.

Kun LN, Ling LW, Wah YW, Lian TT. Epidemiologic study of epilepsy in young Singaporean men. <i>Epilepsia</i>. 1999; 40(10): 1384-7.

Severe Regression

Choudhary A, Gulati S, Sagar R, Kabra M, Sapra S. Behavioral comorbidity in children and adolescents with epilepsy. <i>J Clin Neurosci</i>. 2014; 21(8): 1337-40.

Garcia-Martin G, Perez-Errazquin F, Chamorro-Munoz MI, Romero-Acebal M, Martin-Reyes G, Dawid-Milner MS. Prevalence and clinical characteristics of epilepsy in the South of Spain. <i>Epilepsy Res</i>. 2012; 102(1-2): 100-8.

Zhao Y-H, Zhang Q, Long N, Yang C, Hong J, Mu L, Zhou D. Prevalence of epilepsy and alcohol-related risk in Zayul County, Tibet Autonomous Region in China: an initial survey. <i>Epilepsy Behav</i>. 2010; 19(4): 635-8.

Tse E, Hamiwka L, Sherman EM, Wirrell E. Social skills problems in children with epilepsy: prevalence, nature and predictors. <i>Epilepsy Behav</i>. 2007; 11(4): 499-505.

<p>Debouverie M, Kabore J, Dumas M, Weber M, Duboz P, Vaugelade J. Epidemiology of Epilepsy in Burkina Faso. In: Dumas M, Giordano C, Gentilini M, Chieze F, editors. Neurologie Tropicale. Paris, France: John Libbey Eurotext, 1993. 57-61.</p>

Kwong KL, Chak WK, Wong SN, So KT. Epidemiology of childhood epilepsy in a cohort of 309 Chinese children. <i>Pediatr Neurol</i>. 2001; 24(4): 276-82.

Zhao Y, Zhang Q, Tsering T, Sangwan, Hu X, Liu L, Shang H, Chen Q, Liu Y, Yang X, Wang W, Li S, Wu J, Sander JW, Zhou D. Prevalence of convulsive epilepsy and health-related quality of life of the population with convulsive epilepsy in rural areas of Tibet Autonomous Region in China: an initial survey. <i>Epilepsy Behav</i>. 2008; 12(3): 373-81.

Pascual López MA, Pascual Gispert J, Rodríguez Rivera L, Rojas Ochoa F, Tejeiros A. [Epilepsy: epidemiological study in a child population]. <i>Bol Med Hosp Infant Mex</i>. 1980; 37(4): 811-21.

Del Brutto OH, Santibáñez R, Idrovo L, Rodrìguez S, Díaz-Calderón E, Navas C, Gilman RH, Cuesta F, Mosquera A, Gonzalez AE, Tsang VCW, García HH. Epilepsy and neurocysticercosis in Atahualpa: a door-to-door survey in rural coastal Ecuador. <i>Epilepsia</i>. 2005; 46(4): 583-7.

Cruz-Campos GA, Baquero-Toledo M. [Epilepsies in an outpatient setting: a study of 150 cases]. <i>Rev Neurol</i>. 2000; 30(12): 1108?12.

Josipovic-Jelic Z, Sonicki Z, Soljan I, Demarin V, Collaborative Group for Study of Epilepsy Epidemiology in Sibenik-Knin County, Croatia. Prevalence and socioeconomic aspects of epilepsy in the Croatian county of Sibenik-Knin: community-based survey. <i>Epilepsy Behav</i>. 2011; 20(4): 686-90.

Singh A, Kaur A. Epilepsy in rural Haryana – prevalence and treatment seeking behaviour. <i>J Indian Med Assoc</i>. 1997; 95(2): 37-47.

Cornaggia CM, Canevini MP, Christe W, Giuccioli D, Facheris MA, Sabbadini M, Canger R. Epidemiologic survey of epilepsy among Army draftees in Lombardy, Italy. <i>Epilepsia</i>. 1990; 31(1): 27-32.

Okuma T, Kumashiro H. Natural history and prognosis of epilepsy: report of a multi-institutional study in Japan. The group for the study of prognosis of epilepsy in Japan. <i>Epilepsia</i>. 1981; 22(1): 35-53.

Feksi AT, Kaamugisha J, Gatiti S, Sander JW, Shorvon SD. A comprehensive community epilepsy programme: the Nakuru project. <i>Epilepsy Res</i>. 1991; 8(3): 252-9.

Manonmani V, Tan CT. A study of newly diagnosed epilepsy in Malaysia. <i>Singapore Med J</i>. 1999; 40(1): 32-5.

Waaler PE, Blom BH, Skeidsvoll H, Mykletun A. Prevalence, classification, and severity of epilepsy in children in western Norway. <i>Epilepsia</i>. 2000; 41(7): 802-10.

Simms V, Atijosan O, Kuper H, Nuhu A, Rischewski D, Lavy C. Prevalence of epilepsy in Rwanda: a national cross-sectional survey. <i>Trop Med Int Health</i>. 2008; 13(8): 1047-53.

Mrabet H, Mrabet A, Zouari B, Ghachem R. Health-related quality of life of people with epilepsy compared with a general reference population: a Tunisian study. <i>Epilepsia</i>. 2004; 45(7): 838-

43.

Sahin A, Bolayir E, Sumer H, Tas A, Mollaoglu M, Dener S. Epidemiologic evaluation of epileptic and nonepileptic seizures in Sivas region of middle Anatolia. <i>Neurol Psych Brain Res</i>. 2004; 11(2): 97-102.

Calisir N, Bora I, Irgil E, Boz M. Prevalence of epilepsy in Bursa city center, an urban area of Turkey. <i>Epilepsia</i>. 2006; 47(10): 1691-9.

Chen R-C, Chang Y-C, Chen TH-H, Wu H-M, Liou H-H. Mortality in adult patients with epilepsy in Taiwan. <i>Epileptic Disord</i>. 2005; 7(3): 213-9.

Haerer AF, Anderson DW, Schoenberg BS. Prevalence and clinical features of epilepsy in a biracial United States population. <i>Epilepsia</i>. 1986; 27(1): 66-75.

Nwani PO, Nwosu MC, Asomugha LA, Enwereji KO, Arinzechi EO, Ogunniyi AO. Epidemiology of active epilepsy in a suburban community in Southeast Nigeria: A door-to-door survey. <i>Niger J Clin Pract</i>. 2015; 18(4): 527-33.

Hart YM, Shorvon SD. The nature of epilepsy in the general population. I. Characteristics of patients receiving medication for epilepsy. <i>Epilepsy Res</i>. 1995; 21(1): 43-9.

Tidman L, Saravanan K, Gibbs J. Epilepsy in mainstream and special educational primary school settings. <i>Seizure</i>. 2003; 12(1): 47-51.

Moran NF, Poole K, Bell G, Solomon J, Kendall S, McCarthy M, McCormick D, Nashef L, Sander J, Shorvon SD. Epilepsy in the United Kingdom: seizure frequency and severity, anti-epileptic drug utilization and impact on life in 1652 people with epilepsy. <i>Seizure</i>. 2004; 13(6): 425-33.

Winkler AS, Kerschbaumsteiner K, Stelzhammer B, Meindl M, Kaaya J, Schmutzhard E. Prevalence, incidence, and clinical characteristics of epilepsy--a community-based door-to-door study in northern Tanzania. <i>Epilepsia</i>. 2009; 50(10): 2310-3.

Koul R, Razdan S, Motta A. Prevalence and pattern of epilepsy (Lath/Mirgi/Laran) in rural Kashmir, India. <i>Epilepsia</i>. 1988; 29(2): 116-22.

Treated without Fits Regression

Kong ST, Ho CS, Ho PC, Lim SH. Prevalence of drug resistant epilepsy in adults with epilepsy attending a neurology clinic of a tertiary referral hospital in Singapore. <i>Epilepsy Res</i>. 2014; 108(7): 1253-62.

Fruchter E, Kapara O, Reichenberg A, Yoffe R, Fono-Yativ O, Kreiss Y, Davidson M, Weiser M. Longitudinal association between epilepsy and schizophrenia: a population-based study. <i>Epilepsy Behav</i>. 2014; 31: 291-4.

Tanaka A, Akamatsu N, Shouzaki T, Toyota T, Yamano M, Nakagawa M, Tsuji S. Clinical characteristics and treatment responses in new-onset epilepsy in the elderly. <i>Seizure</i>. 2013; 22(9): 772-5.

Nickels KC, Grossardt BR, Wirrell EC. Epilepsy-related mortality is low in children: a 30-year population-based study in Olmsted County, MN. <i>Epilepsia</i>. 2012; 53(12): 2164-71.

Picot M-C, Baldy-Moulinier M, Daurès J-P, Dujols P, Crespel A. The prevalence of epilepsy and pharmacoresistant epilepsy in adults: a population-based study in a Western European country. <i>Epilepsia</i>. 2008; 49(7): 1230-8.

Tse E, Hamiwka L, Sherman EM, Wirrell E. Social skills problems in children with epilepsy: prevalence, nature and predictors. <i>Epilepsy Behav</i>. 2007; 11(4): 499-505.

Ding D, Hong Z, Chen GS, Dai XY, Wu JZ, Wang WZ, De Boer HM, Sander JW, Prilipko L, Chisholm D. Primary care treatment of epilepsy with phenobarbital in rural China: Cost-outcome analysis from the WHO/ILAE/IBE global campaign against epilepsy demonstration project. <i>Epilepsia</i>. 2008; 49(3): 535-9.

Houinato D, Yemadje L-P, Glitho G, Adjien C, Avode G, Druet-Cabanac M, Preux P-M. Epidemiology of epilepsy in rural Benin: prevalence, incidence, mortality, and follow-up. <i>Epilepsia</i>. 2013; 54(4): 757-63.

Hunter E, Rogathi J, Chigudu S, Jusabani A, Jackson M, Whittaker RG, Gray W, McNally RJ, Aris E,

Mushi D, Walker R. The epilepsy treatment gap in rural Tanzania: A community-based study in adults. <i>Seizure</i>. 2016; 36: 49-56.
Kun LN, Ling LW, Wah YW, Lian TT. Epidemiologic study of epilepsy in young Singaporean men. <i>Epilepsia</i>. 1999; 40(10): 1384-7.

Treatment Gap Regression

Huang DH, Zheng JO, Chen J, Yu L. Treatment gaps of epilepsy and retention rates of sodium valproate in rural Guangxi, China. <i>Genet Mol Res</i>. 2014; 13(3): 6202-12.
Pandey S, Singhi P, Bharti B. Prevalence and treatment gap in childhood epilepsy in a north Indian city: a community-based study. <i>J Trop Pediatr</i>. 2014; 60(2): 118-23.
Farghaly WM, El-Tallawy HN, Rageh TA, Mohamed EM, Metwally NA, Shehata GA, Badry R, Abd-Elhamed MA. Epidemiology of uncontrolled epilepsy in the Al-Kharga District, New Valley, Egypt. <i>Seizure</i>. 2013; 22(8): 611-6.
Hunter E, Rogathi J, Chigudu S, Jusabani A, Jackson M, McNally R, Gray W, Whittaker RG, Iqbal A, Birchall D, Aris E, Walker R. Prevalence of active epilepsy in rural Tanzania: a large community-based survey in an adult population. <i>Seizure</i>. 2012; 21(9): 691-8.
Mbuba CK, Ngugi AK, Fegan G, Ibinda F, Muchohi SN, Nyundo C, Odhiambo R, Edwards T, Odermatt P, Carter JA, Newton CR. Risk factors associated with the epilepsy treatment gap in Kilifi, Kenya: a cross-sectional study. <i>Lancet Neurol</i>. 2012; 11(8): 688-96.
Malik MA, Akram RM, Tarar MA, Sultan A. Childhood epilepsy. <i>J Coll Physicians Surg Pak</i>. 2011; 21(2): 74-8.
Zhao Y-H, Zhang Q, Long N, Yang C, Hong J, Mu L, Zhou D. Prevalence of epilepsy and alcohol-related risk in Zayul County, Tibet Autonomous Region in China: an initial survey. <i>Epilepsy Behav</i>. 2010; 19(4): 635-8.
Nicoletti A, Sofia V, Vitale G, Bonelli SI, Bejarano V, Bartalesi F, Tran DS, Preux PM, Zappia M, Bartoloni A. Natural history and mortality of chronic epilepsy in an untreated population of rural Bolivia: a follow-up after 10 years. <i>Epilepsia</i>. 2009; 50(10): 2199-206.
Kobau R, Zahran H, Grant D, Thurman DJ, Price PH, Zack MM. Prevalence of active epilepsy and health-related quality of life among adults with self-reported epilepsy in California: California Health Interview Survey, 2003. <i>Epilepsia</i>. 2007; 48(10): 1904-13.
Noronha ALA, Borges MA, Marques LHN, Zanetta DMT, Fernandes PT, de Boer H, Espíndola J, Miranda CT, Prilipko L, Bell GS, Sander JW, Li LM. Prevalence and pattern of epilepsy treatment in different socioeconomic classes in Brazil. <i>Epilepsia</i>. 2007; 48(5): 880-5.
Oun A, Haldre S, Magi M. Use of antiepileptic drugs in Estonia: an epidemiologic study of adult epilepsy. <i>Eur J Neurol</i>. 2006; 13(5): 465-70.
Kochen S, Melcon MO. Prognosis of epilepsy in a community-based study: 8 years of follow-up in an Argentine community. <i>Acta Neurol Scand</i>. 2005; 112(6): 370-4.
Somoza MJ, Forlenza RH, Brussino M, Licciardi L. Epidemiological survey of epilepsy in the primary school population in Buenos Aires. <i>Neuroepidemiology</i>. 2005; 25(2): 62-8.
Gomes Md M da M, Zeitoune RG, Kropf LAL, Beeck Ed E da S van. A house-to-house survey of epileptic seizures in an urban community of Rio de Janeiro, Brazil. <i>Arq Neuropsiquiatr</i>. 2002; 60(3-B): 708-11.
Wang W, Wu J, Wang D, Chen G, Wang T, Yuan C, Yang B, Zhao D. [Epidemiological survey on epilepsy among rural populations in five provinces in China]. <i>Nat Med J Chin</i>. 2002; 82(7): 449-52.
Zhao Y, Zhang Q, Tsering T, Sangwan, Hu X, Liu L, Shang H, Chen Q, Liu Y, Yang X, Wang W, Li S, Wu J, Sander JW, Zhou D. Prevalence of convulsive epilepsy and health-related quality of life of the population with convulsive epilepsy in rural areas of Tibet Autonomous Region in China: an initial survey. <i>Epilepsy Behav</i>. 2008; 12(3): 373-81.
Placencia M, Shorvon SD, Paredes V, Bimos C, Sander JW, Suarez J, Cascante SM. Epileptic seizures in an Andean region of Ecuador. Incidence and prevalence and regional variation. <i>Brain</i>. 1992; 115 (Pt 3): 771-82.

Del Brutto OH, Santibáñez R, Idrovo L, Rodrìguez S, Díaz-Calderón E, Navas C, Gilman RH, Cuesta F, Mosquera A, Gonzalez AE, Tsang VCW, García HH. Epilepsy and neurocysticercosis in Atahualpa: a door-to-door survey in rural coastal Ecuador. <i>Epilepsia</i>. 2005; 46(4): 583-7.
Benavente I, Rubio E, Morales C, Tajada N, Tamargo P. Prevalence of epilepsy amongst adolescents in Huesca, Spain: a community-based study. <i>Eur J Neurol</i>. 2009; 16(10): 1138-43.
Tekle-Haimanot R, Forsgren L, Ekstedt J. Incidence of epilepsy in rural central Ethiopia. <i>Epilepsia</i>. 1997; 38(5): 541-6.
Almu S, Tadesse Z, Cooper P, Hackett R. The prevalence of epilepsy in the Zay Society, Ethiopia – an area of high prevalence. <i>Seizure</i>. 2006; 15(3): 211-3.
Löfgren E, Pouta A, von Wendt L, Tapanainen J, Isojärvi JIT, Järvelin M-R. Epilepsy in the northern Finland birth cohort 1966 with special reference to fertility. <i>Epilepsy Behav</i>. 2009; 14(1): 102-7.
Jallon P. [Evaluation of the prevalence of epilepsy in a military selection centre]. <i>Rev Neurol (Paris)</i>. 1991; 147(4): 319-22.
Ross EM, Peckham CS, West PB, Butler NR. Epilepsy in childhood: findings from the National Child Development Study. <i>BMJ</i>. 1980; 280(6209): 207-10.
Coleman R, Loppy L, Walraven G. The treatment gap and primary health care for people with epilepsy in rural Gambia. <i>Bull World Health Organ</i>. 2002; 80(5): 378-83.
Medina MT, Durón RM, Martínez L, Osorio JR, Estrada AL, Zúniga C, Cartagena D, Collins JS, Holden KR. Prevalence, incidence, and etiology of epilepsies in rural Honduras: the Salama Study. <i>Epilepsia</i>. 2005; 46(1): 124-31.
Koul R, Razdan S, Motta A. Prevalence and pattern of epilepsy (Lath/Mirgi/Laran) in rural Kashmir, India. <i>Epilepsia</i>. 1988; 29(2): 116-22.
Mani KS, Rangan G, Srinivas HV, Kalyanasundaram S, Narendran S, Reddy AK. The Yelandur study: a community-based approach to epilepsy in rural South India – epidemiological aspects. <i>Seizure</i>. 1998; 7(4): 281-8.
Saha SP, Bhattacharya S, Das SK, Maity B, Roy T, Raut DK. Epidemiological study of neurological disorders in a rural population of Eastern India. <i>J Indian Med Assoc</i>. 2003; 101(5): 299-304.
Hackett RJ, Hackett L, Bhakta P. The prevalence and associated factors of epilepsy in children in Calicut District, Kerala, India. <i>Acta Paediatr</i>. 1997; 86(11): 1257-60.
Banerjee TK, Ray BK, Das SK, Hazra A, Ghosal MK, Chaudhuri A, Roy T, Raut DK. A longitudinal study of epilepsy in Kolkata, India. <i>Epilepsia</i>. 2010; 51(12): 2384-91.
Sureka RK, Sureka R. Prevalence of epilepsy in rural Rajasthan--a door-to-door survey. <i>J Assoc Physicians India</i>. 2007; 55: 741-2.
Edwards T, Scott A, Munyoki G, Odera V, Chengo E, Bauni E, Kwasa T, Sander L, Neville B, Newton C. Active convulsive epilepsy in a rural district of Kenya: a study of prevalence and possible risk factors. <i>Lancet Neurol</i>. 2008; 7(1): 50-6.
Tran D-S, Odermatt P, Le T-O, Huc P, Druet-Cabanac M, Barennes H, Strobel M, Preux P-M. Prevalence of epilepsy in a rural district of central Lao PDR. <i>Neuroepidemiology</i>. 2006; 26(4): 199-206.
Traore M, Tahny R, Sacko M. Prévalence de l'épilepsie chez les enfants de 3 à 15 ans dans 2 communes du district de Bamako. <i>Rev Neurol (Paris)</i>. 2000; 156(Suppl 1): S18.
Brodtkorb E, Sjaastad O. Epilepsy prevalence by individual interview in a Norwegian community. <i>Seizure</i>. 2008; 17(7): 646-50.
Svendsen T, Lossius M, Nakken KO. Age-specific prevalence of epilepsy in Oppland County, Norway. <i>Acta Neurol Scand</i>. 2007; 116(5): 307-11.
Aziz H, Güvener A, Akhtar SW, Hasan KZ. Comparative epidemiology of epilepsy in Pakistan and Turkey: population-based studies using identical protocols. <i>Epilepsia</i>. 1997; 38(6): 716-22.
Ndoye NF, Sow AD, Diop AG, Sessouma B, Séne-Diouf F, Boissy L, Wone I, Touré K, Ndiaye M, Ndiaye P, de Boer H, Engel J, Mandlhate C, Meinardi H, Prilipko L, Sander JWAS. Prevalence of epilepsy its treatment gap and knowledge, attitude and practice of its population in sub-urban Senegal an ILAE/IBE/WHO study. <i>Seizure</i>. 2005; 14(2): 106-11.
Karaagaç N, Yeni SN, Senocak M, Bozluolçay M, Savrun FK, Ozdemir H, Cagatay P. Prevalence of

epilepsy in Silivri, a rural area of Turkey. <i>Epilepsia</i>. 1999; 40(5): 637-42.

Topalkara K, Akyuz A, Sumer H, Bekar D, Topaktas S, Dener S. An epilepsy prevalence study performed using a stratified sampling method among urban residents of Sivas. <i>Epilepsi</i>. 1999; 5(1): 24-9.

Sahin A, Bolayir E, Sumer H, Tas A, Mollaoglu M, Dener S. Epidemiologic evaluation of epileptic and nonepileptic seizures in Sivas region of middle Anatolia. <i>Neurol Psych Brain Res</i>. 2004; 11(2): 97-102.

Calisir N, Bora I, Irgil E, Boz M. Prevalence of epilepsy in Bursa city center, an urban area of Turkey. <i>Epilepsia</i>. 2006; 47(10): 1691-9.

Su C, Chang S, Chen Z, Lee C, Chen R. Neuroepidemiological survey in Ilan, Taiwan (NESIT): (4) Prevalence of epilepsy. <i>Acta Neurol Taiwan</i>. 1998; 7(2): 75-84.

Dent W, Helbok R, Matuja WBP, Scheunemann S, Schmutzhard E. Prevalence of active epilepsy in a rural area in South Tanzania: a door-to-door survey. <i>Epilepsia</i>. 2005; 46(12): 1963-9.

Winkler AS, Kerschbaumsteiner K, Stelzhammer B, Meindl M, Kaaya J, Schmutzhard E. Prevalence, incidence, and clinical characteristics of epilepsy--a community-based door-to-door study in northern Tanzania. <i>Epilepsia</i>. 2009; 50(10): 2310-3.

Chiang KL, Cheng CY. Prevalence and neuro-psychiatric comorbidities of pediatric epilepsy in Taiwan: a national population-based study. <i>Epilepsy Res</i>. 2014; 108(8): 1451-60.

Khedr EM, Shawky OA, Ahmed MA, Elfetoh NA, Al Attar G, Ali AM, Kandil MR, Farweez H. A community based epidemiological study of epilepsy in Assiut Governorate/Egypt. <i>Epilepsy Res</i>. 2013; 103(3-Feb): 294-302.

Hu J, Si Y, Zhou D, Mu J, Li J, Liu L, Zhu CR, Deng Y, He J, Zhang NM, Chen XF. Prevalence and treatment gap of active convulsive epilepsy: a large community-based survey in rural West China. <i>Seizure</i>. 2014; 23(5): 333-7.

Nwani PO, Nwosu MC, Enwereji KO, Asomugha AL, Arinzechi EO, Ogunniyi AO. Epilepsy treatment gap: prevalence and associated factors in Southeast Nigeria. <i>Acta Neurol Scand</i>. 2013; 128(2): 83-90.

El-Tallawy HN, Farghaly WM, Shehata GA, Abdel-Hakeem NM, Rageh TA, Abo-Elftoh NA, Hegazy A, Badry R. Epidemiology of epilepsy in New Valley Governorate, Al Kharga District, Egypt. <i>Epilepsy Res</i>. 2013; 104(2-Jan): 167-74.

Pi X, Zhou L, Cui L, Liu A, Zhang J, Ma Y, Liu B, Cai C, Zhu C, Zhou T, Chen J, Zhou Z, Wang C, Li L, Li S, Wu J, Xiao B. Prevalence and clinical characteristics of active epilepsy in southern Han Chinese. <i>Seizure</i>. 2014; 23(8): 636-40.

Lomidze G, Kasradze S, Kvernadze D, Okujava N, Toidze O, de Boer HM, Dua T, Sander JW. The prevalence and treatment gap of epilepsy in Tbilisi, Georgia. <i>Epilepsy Res</i>. 2012; 98(2-3): 123-9.

Goel D, Dhanai JS, Agarwal A, Mehlotra V, Saxena V. Neurocysticercosis and its impact on crude prevalence rate of epilepsy in an Indian community. <i>Neurol India</i>. 2011; 59(1): 37-40.

Velioglu SK, Bakirdemir M, Can G, Topbas M. Prevalence of epilepsy in northeast Turkey. <i>Epileptic Disord</i>. 2010; 12(1): 22-37.

El-Tallawy HN, Farghaly WM, Rageh TA, Shehata GA, Metwally NA, Badry R, Sayed MA, Abdelwarith AM, Kandil MR, Hamed MA, Mohamed KO, Tohamy AM. Spectrum of epilepsy - prevalence, impact, and treatment gap: an epidemiological study from Al-Quseir, Egypt. <i>Neuropsychiatr Dis Treat</i>. 2016; 12: 1111-8.

Hunter E, Rogathi J, Chigudu S, Jusabani A, Jackson M, Whittaker RG, Gray W, McNally RJ, Aris E, Mushi D, Walker R. The epilepsy treatment gap in rural Tanzania: A community-based study in adults. <i>Seizure</i>. 2016; 36: 49-56.

Serrano-Castro PJ, Mauri-Llerda JA, Hernandez-Ramos FJ, Sanchez-Alvarez JC, Parejo-Carbonell B, Quiroga-Subirana P, Vazquez-Gutierrez F, Santos-Lasaosa S, Mendez-Lucena C, Redondo-Verge L, Tejero-Juste C, Morandeira-Rivas C, Sancho-Rieger J, Matias-Guiu J. Adult Prevalence of Epilepsy in Spain: EPIBERIA, a Population-Based Study. <i>ScientificWorldJournal</i>. 2015; 2015: 602710.

Hashem S, Al-Kattan M, Ibrahim SY, Shalaby NM, Shamloul RM, Farrag M. Epilepsy prevalence in Al-Manial Island, Egypt. A door-to-door survey. <i>Epilepsy Res</i>. 2015; 117: 133-7.

Sebera F, Munyandamutsa N, Teuwen DE, Ndiaye IP, Diop AG, Tofighy A, Boon P, Dedeken P. Addressing the treatment gap and societal impact of epilepsy in Rwanda--Results of a survey conducted in 2005 and subsequent actions. <i>Epilepsy Behav</i>. 2015; 46: 126-32.
Banerjee TK, Dutta S, Ray BK, Ghosal M, Hazra A, Chaudhuri A, Das SK. Epidemiology of epilepsy and its burden in Kolkata, India. <i>Acta Neurol Scand</i>. 2015; 132(3): 203-11.
**Wang WZ, Wu JZ, Wang DS, Dai XY, Yang B, Wang TP, Yuan CL, Scott RA, Prilipko LL, de Boer HM, Sander JW.** The prevalence and treatment gap in epilepsy in China: an ILAE/IBE/WHO study. <i>Neurology</i>. 2003; 60(9): 1544–5.
Nizamie SH, Akthar S, Banerjee I, Goyal N. Health care delivery model in epilepsy to reduce treatment gap: World Health Organization study from a rural tribal population of India. <i>Epilepsy Res</i>. 2009; 84(2–3): 146–52.
Banerjee TK, Hazra A, Biswas A, Ray J, Roy T, Raut DK, Chaudhuri A, Das SK. Neurological disorders in children and adolescents. <i>Indian J Pediatr</i>. 2009; 76(2): 139-46.
Goel D, Agarwal A, Dhanai JS, Semval VD, Mehrotra V, Saxena V, Maithili B. Comprehensive rural epilepsy surveillance programme in Uttarakhand state of India. <i>Neurol India</i>. 2009; 57(3): 355–6.
Pal DK, Das T, Sengupta S. Comparison of key informant and survey methods for ascertainment of childhood epilepsy in West Bengal, India. <i>Int J Epidemiol</i>. 1998; 27(4): 672–6.
Levy LF. Epilepsy in Rhodesia, Zambia, and Malawi. <i>Afr J Med Sci</i>. 1970; 1( ): 291-203.
Kun LN, Ling LW, Wah YW, Lian TT. Epidemiologic study of epilepsy in young Singaporean men. <i>Epilepsia</i>. 1999; 40(10): 1384-7.
